# 1,1′-Disubstituted Ferrocene Ligand Scaffolds Featuring Pnictogens Other than Phosphorus as Donor Sites

**DOI:** 10.3390/molecules29225283

**Published:** 2024-11-08

**Authors:** Subhayan Dey, Rudolf Pietschnig

**Affiliations:** 1 Department of Chemistry, School of Advanced Sciences, Vellore Institute of Technology, Vandalur-Kelambakkam Road, Chennai 600127, Tamil Nadu, India; 2 Institut für Chemie und CINSaT, University of Kassel, Heinrich Plett-Straße 40, 34132 Kassel, Germany

**Keywords:** pnictogens, ferrocene, dppf-analogs, amine, arsenic, antimony, bismuth, homoditopic ligands, heteroditopic ligands

## Abstract

The chemistry of bidentate ligands with a dppf-like motif, where phosphorus is fully or partially replaced by other pnictogens as donor sites, is summarized and discussed in this comprehensive review, while covering the literature from 1966 to 2024, related to more than 165 original references and discussing more than 75 independent chemical entities (**1**–**41**). Besides addressing synthetic, structural, and electrochemical aspects of such compounds, their donor properties and metal coordination behavior is discussed, along with catalytic applications. Based on their electronic and steric situations, trends in the performance of such compounds, either as ligands for catalysis or on their own merits for non-catalytic purposes, have been elucidated. Related topics that could not be covered in this article have been acknowledged by referring to the literature for completeness.

## 1. Introduction

**Figure 1:**
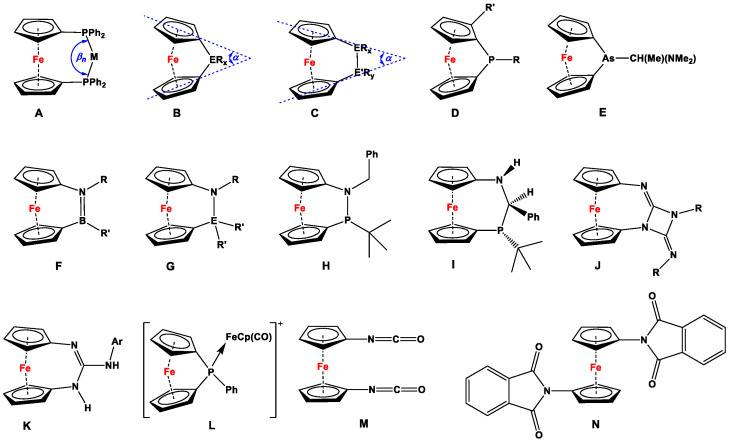
Bite angle *β_n_* for a chelated complex of dppf (**A**) [7]; [1]FCP and its dihedral angle *α* (**B**) [16]; [2]FCP and its dihedral angle *α* (**C**) [23,24]; phospha [1]FCP, where R = Ph, CH(Me)(NMe_2_) and R’ = N*^i^*Pr_2_, Ph, *^t^*Bu, Cl, etc. (**D**) [15]; arsa [1]FCP (**E**) [15]; azabora [2]FCP (**F**) [25]; aza [2]FCP, where ER’_2_ = SiMe_2_, Sn*^t^*Bu_2_, Si*^t^*Bu_2_, and R = SiMe_3_ (**G**) [23]; azaphospha [2]FCP (**H**) [24]; azacarbaphospha [3]FCP (**I**) [24]; diazacarba [3]FCP with paramagnetic substituents (**J**) [26,28,29,30]; arylamino-1,3-diaza [3]ferrocenophanes (**K**) [26]; intermediate for photoinduced ROP of **D** (**L**) [15,27]; 1,1′-diisocyanatoferrocene (**M**) [31,32,33]; and 1,1′-dipthalimidoferrocene (**N**) [34,35].

By hosting mono- [1], di- [2], tri- [3,4], and multidentate phosphanyl ligand systems [5], ferrocene has played a vital role in complexation and catalysis for almost six decades. Despite the dominance of dppf (i.e., 1,1′-bis(diphenylphosphino)ferrocene or 1,1′-bis(diphenylphosphanyl)ferrocene according to IUPAC) [6] and its slimmer and bulkier counterparts as bidentate ligands [7], related 1,1′-bischalcogen and 1,1′-bispnictogen ligands have further emerged over time [8,9,10]. Unlike other bidentate ligands with alkyl (e.g., 1,2-bis(diphenylphosphino)ethane) and alkenyl (e.g., *cis*-1,2-bis(diphenylphosphino)ethylene) spacers, ferrocene provides a robust yet flexible backbone, which allows a variety of metal centers to be stabilized by attaining various facile spatial orientations (such as classical chelated; open-bridged; double-bridged; quasi-closed bridged; *η*^1^, *η*^1^-intrabridged; *η*^1^, *η*^1^-interbridged; and quasi-closed double-bridged complexes) [7,11,12]. At the same time, the 1,1′-(bisphosphino)ferrocene ligand family shows higher bite angles (*β_n_*, Figure 1A) during complexation [13], which further plays an instrumental role in catalysis, usually resulting in higher conversion rates than comparable reactions with their alkyl and alkenyl counterparts [14].

Other than contributing to ligand chemistry and catalysis, pnictogen-substituted ferrocenylene species further constitute a major subsection of ferrocenophanes (FCPs, Figure 1B,C), where the resulting rings feature moderate to high molecular strain [15]. Dihedral angle *α* is considered the key parameter of molecular deformation, which has been related to ring strain and thermodynamic aspects of ring-opening polymerization (ROP) reactions [16], allowing different compounds to be compared. Following the previously demonstrated trends, the *α* angle decreases with the increase in the size and number of bridging atoms [15,17,18]. In this respect, the largest *α* angle is manifested by P-bridged [1]FCPs, for which the values vary narrowly between 26.9 and 27.9°, depending upon the nature of the substituents on phosphorus and in the α-positions of the ferrocene (Figure 1D) [15]. Owing to the bigger atomic size of arsenic, the *α* angle of arsa [1]FCP (*α* = 22.9°) is significantly smaller than those of phospha [1]FCPs (Figure 1E) [19].

Besides [1]FCPs, there are a handful of examples reported for pnictogen-bridged [*n*] FCPs (*n* = 2, 3) [17,18,20,21,22,23,24], among which the highest dihedral angles were found for a family of B,N-bridged [2]FCPs (*α* = 22.9–24.2°), where the bridging B=N bonds can further be considered isoelectronic to the C=C bond (Figure 1F) [25]. On the other hand, N,Si and N,Sn [2]FCPs (Figure 1G) showed low to moderate dihedral angles (*α* = 9.36–15.73°), and therefore, no detectable polymers were observed after ROP reactions [23]. N,P [2]FCP H demonstrates a rare example for mixed pnictogen-bridged [2]FCP, which, upon prolonged standing at room temperature, isomerizes into N,C,P [3]FCP I, accompanied by a decrease in the *α* angle (from 17.93–18.15° for H to 5.81–9.49° for I), which is a determining factor in transforming [2]FCP to [3]FCP [24]. Upon replacement of P in the *ansa*-position with a smaller pnictogen-like N, diazacarba [3]FCP with paramagnetic (J, *α* = 11.80–14.31°) and arylamino- (K, *α* = 15.57–16.44°) substituents showed considerably higher dihedral angles [26] compared to alkylidene-bridged aminophosphanyl [3]FCP I (*α* = 5.81–9.49°) [24]. However, none of the compounds D–K have been used for complexation and catalysis, except for photoinduced ROP of D with R = Ph and R’ = H, where the ring opening reaction is believed to proceed via in situ formation of a complex L [15,27].

**Scheme 1:**
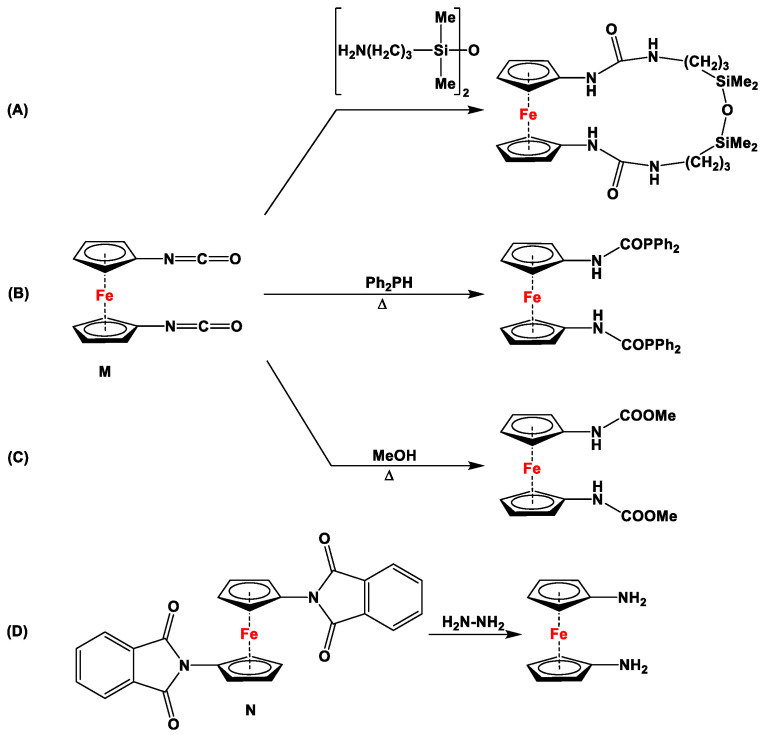
A few selected reactions with compounds **M** (**A**–**C**) and **N** (**D**) [32,35,43,45,46].

Although several excellent review articles and book chapters discuss syntheses and catalytic features of dppf and its analogs [7,8,11,36,37,38,39,40,41], to the best of our knowledge, there are no reports available that solely concentrate on the recent advancements in their non-phosphanyl counterparts. Unlike several bisphosphanyl-substituted dppf analogs, where detailed computational assessment on the bite angles *β_n_* and catalytic activities have frequently been reported [7,42], such data are unavailable for their (N,N), (As,As), (Sb,Sb), (Bi,Bi), or (N,P) counterparts. As they are lacking pronounced chemical applications, 1,1′-diisocyanato- (Figure 1M) and 1,1′-dipthalimidoferrocene (Figure 1N) have not been put into focus here. Compounds **M** and **N** have notably been used as starting materials to functionalize ferrocenes with amino and (oxycarbonyl)amino moieties via reduction with other amines (for **M**, Scheme 1) [31,32,33], phosphines (for **M**, Scheme 1) [43], and alcohols (for **M**, Scheme 1) [44,45], and via Gabriel-type synthesis with hydrazine (for **N**, Scheme 1) [34,35].

For a better overview, the wealth of 1,1′-bispnictogen-substituted dppf analogs has been divided into two major categories: 1,1′-symmetrically and -unsymmetrically substituted systems. 1,1′-Symmetrically substituted compounds are discussed depending on their substituents on ferrocenes, and therefore, have further been classified as 1,1′-diamino- (**1**–**12**), 1,1′-diimidazolium- (**13**–**16**), 1,1′-diimino- (**17**–**19**), 1,1′-diarsanyl- (**20**–**23**), 1,1′-distibanyl- (**24**), and 1,1′-dibismuthanyl ferrocenes (**25** and **26**). On the other hand, 1,1′-unsymmetrically substituted systems are subdivided into the following three groups: 1,1′-N,P (**27**–**39**), 1,1′-arsanylphosphanyl- (**40**), and 1,1′-arsanylstibanylferrocenes (**41**). Owing to the sake of simplicity and their low abundance, multiferrocenyl and multidentate ligand systems are kept out of our discussion, and consequently, readers interested in such ligands are referred to specialized articles for further information [47,48].

## 2. Itemization and Inventory

Although the main text of the current article summarizes 1,1′-pnictogen-disubstituted dppf analogs, their chemical structures, synthetic precursors, respective complexes, and applications have been listed in Table 1 for a better overview. Table 1 further serves the purpose of synoptical documentation, so that the functional details of **1**–**38** can be found via a quick and easy inspectional survey, without investing much time in comprehensive reading.

**Table 1:** Overview of the synthetic access and chemical uses for 1,1′-bispnictogen-substituted dppf analogs.

Group 15 Elements Substituting 1,1′-Ferrocenes	Precursors for Syntheses	Chemical Applications and Complexes
*1,1′-Symmetrically substituted systems: 1,1′-diaminoferrocenes*		
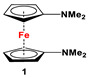	C_5_H_4_LiNMe_2_ and FeCl_2_ (Scheme 2A) [49]	Isolated complexes of **1** are reported with TiCl_2_ [50]. Compound **1** has frequently been used for the purpose of electrochemical measurements [51,52,53].
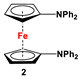	Fc’Br_2_, NaNPh_2_, and CuI [54]	Compound **2** has been used for electrochemical measurements [51,53,54].
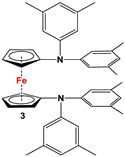	Fc’(NH_2_)_2_, 3,5-Me_2_-C_6_H_3_-Br, and PdBINAP [55]	No application reported [55].
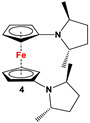	Fc’(NH_2_)_2_ and (*2R*,*5R*)-2,5-hexanediol cyclic sulfate [56]	Used for comprehending N-Cp^Fc^ electron donation [56].
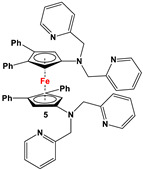	C_5_H_4_LiN(CH_2_Py)_2_ and FeCl_2_ (Scheme 2B) [57]	Isolated complex of **5** is reported with ZnBr_2_, Zn(CF_3_SO_3_)_2_, and Co(CF_3_SO_3_)_2_ [58,59,60]. Compound **5** has further been used for synthesizing redox-switchable complexes.
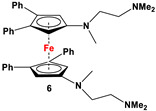	C_5_H_4_LiNMe(CH_2_CH_2_NMe_2_) and FeCl_2_ (Scheme 2B) [57]	Compound **6** has been used for electrochemical measurements [58].
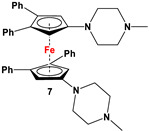	C_5_H_4_LiN(CH_2_)_4_NMe and FeCl_2_ (Scheme 2B) [57,58]	Compound **7** was used for electrochemical measurements [58].
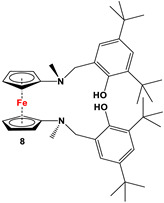	Fc’(NH_2_)_2_ and 3,5-di-*tert*-butyl-2-hydroxybenzaldehyde [61].	Isolated complexes of **8** are reported with Zr(O*^t^*Bu)_2_, which was further used in ROP for L-lactide and ε-caprolactone [61].
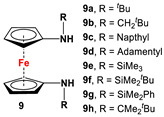	Fc’(NH_2_)_2_ and aldehydes or silylchlorides or respective ketones (with *p*-toluene sulfonic acid monohydrate) [62,63,64,65,66].	Different variations of **9** were used for electrochemical measurements and computational purposes [64], and to act as substituents for carbenes, stannylenes, germylenes [62,63,67], Zr(Bn)_2_, Mg(THF)_2_, TiCl_2_, and TiMe_2_ [68]. Germylenes with deprotonated **9a**, **9d**, **9e**, and **9h** were further explored for oxidation reactions with S, Se, and (PhSe)_2_ [69]. Isolated complexes of [M(CH_2_Ar)(THF)] (M = Sc, Y, La, Lu) with **9d** and **9f** were used for dearomatization and ring-opening reactions [70,71,72,73,74].
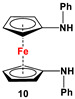	Fc’(NH_2_)_2_, PhBr, and Pd_2_(dba)_3_ (similar to Scheme 2D) [75].	Compound **10** was used to synthesize zirconium chelates [76].
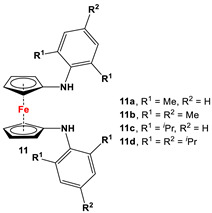	Fc’(NH_2_)_2_, respective arylbromides, and Pd_2_(dba)_3_ (similar to Scheme 2D) [65,75,77].	**11b** and **11c** were used to synthesize N-heterocyclic silylenes [78], germylenes, and stannylenes [79]. Germylenes of **11b** and **11c** were further explored for oxidation reactions with S, Se, and (PhSe)_2_ [69]. Isolated complexes of **11c** were reported with Al(III) [77]. Isolated complexes of **11d** were reported with Zr(NMe_2_)_2_ and Zr(Bz)_2_ [76].
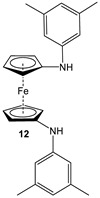	Fc’(NH_2_)_2_, 3,5-Me_2_-C_6_H_3_-Br, and PdBINAP [55].	No application reported [55].
*1,1′-Symmetrically substituted systems: 1,1′-diimidazoliumferrocenes*		
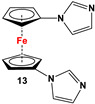	Fc’I_2_, imidazole and CuI [80].	Compound **13** was used to synthesize ferrocene-based redox-responsive receptors [80].
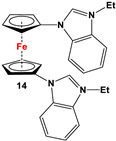	Fc’(NH_2_)_2_ and 2-fluoronitrobenzene [81].	Isolated complexes of compound **14** were reported with Ir(cod), where “cod” stands for 1,5-cyclooctadiene [81].
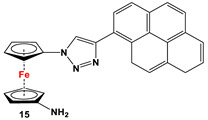	Fc’(N_3_)_2_, ethynylpyrene and CuSO_4_ [82].	Compound **15** was used to synthesize ion-pair recognition receptors [82].
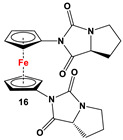	**M** and L-proline (Scheme 2E) [31].	No application reported [31].
*1,1′-Symmetrically substituted systems: 1,1′-diiminoferrocenes*		
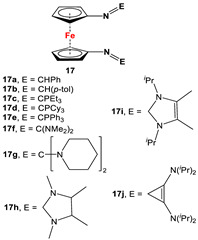	Fc’(NH_2_)_2_ and respective arylaldehydes [83,84,85,86,87].	Isolated complex of **17a** was reported with PdCl_2_ and PdMeCl_,_ which have further been used for catalytic purposes [88,89]. Reduced versions of **17** were used for complexation with Zr(Bz)_4_ [90]. Cationic Ni(II) and Pd(II) complexes are reported with **17c**–**17i** [84,85,86,87].
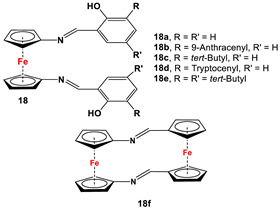	Fc’(NH_2_)_2_ and respective arylaldehydes (for **18a**–**18e**) [89,91]; Fc’(N_3_)_2_, PPh_3_, and Fc’(CHO)_2_ (for **18f**) [92].	Isolated complexes for different variations of **18** were reported with Zr(Bz)_2_ [91], Mg(THF)_2_ [91], TiCl_2_ [89], Ti(O*^i^*Pr)_2_ [89,93,94], Ce(O*^t^*Bu)_2_ [95], In(O*^t^*Bu) [94,96], Zn [97], Co [97], Zr(O*^i^*Pr)_2_ [98], Zr(O*^t^*Bu)_2_ [94,98], and Al(O*^i^*Pr) [94]. Some of these complexes were further used for ethylene, lactone, and lactide polymerizations [89,93,94,95,96,98].
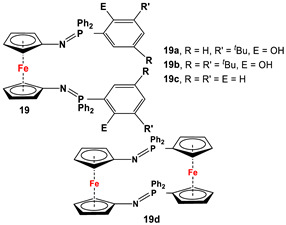	Fc’(N_3_)_2_ and respective arylphosphine (Scheme 2F) [92,99,100].	Isolated complexes with 19 are reported with Ce(O*^t^*Bu)_2_ [99], Ce(O*^t^*Bu)THF [99], CeCl(THF) [99], CeI(THF) [99], YCl [99], Y(O*^t^*Bu) [99], YCl [101], Y(CH_2_Ph) [101], and Y(CH_2_SiMe_3_) [101].
*1,1′-Symmetrically substituted systems: 1,1’-diarsanylferrocenes*		
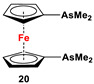	Fc’Li_2_·2/3tmeda and Me_2_AsCl (Scheme 3A) [2].	Isolated complexes with compounds **20** are reported with Cr(CO)_4_ [102], Mo(CO)_4_ [102], W(CO)_4_ [102], PdCl_2_ [103], PdBr_2_ [103], PtCl_2_ [103], PtBr_2_ [103], PtI_2_ [103], Ni(CO)_2_ [103], Ni(CO)I_2_ [103], NiBr_2_ [104], and [Cu(MeCN)_2_]BF_4_ [105].
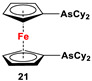	1,1′-bis(benzodithiaarsole) ferrocene and excess CyMgCl (Scheme 3E) [106].	**21** was reported for in situ complexation with Pd_2_(dba)_3_, which was further used as an arsa-Buchwald ligand for catalytic purposes [106].
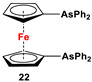	Fc’Li_2_·2/3tmeda and Ph_2_AsCl (Scheme 3A) [2].	Isolated complexes with compounds **20** are reported with Cr(CO)_4_ [102], Mo(CO)_4_ [102], W(CO)_4_ [102], Ni(CO)_2_ [103], Ni(CO)I_2_ [103], (*η*^2^-C_60_)Pt [107], and PdCl_2_ [108].
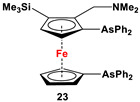	(*α*-CH_2_NMe_2_, *β*-SiMe_3_-C_5_H_3_)(C_5_H_5_)Fe, *^n^*BuLi, and Ph_2_AsCl (Scheme 3C) [109].	Fungicidal activity of compound **23** for crop plants was examined against fusarium head blight of wheat, early blight of tomato, wilt disease of cotton, ring-rot disease of apple, and brown blotch disease of peanut [109].
*1,1′-Symmetrically substituted systems: 1,1′-distibanylferrocene*		
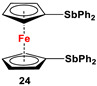	Fc’Br_2_, *^n^*BuLi, and Ph_2_SbCl (Scheme 3B) [110].	Isolated complexes with compounds **24** are reported with AgClO_4_ [110]. Compound **24** was further oxidized to stiboranes, which was eventually converted to a rare SbOSb [3]FCP [110].
*1,1′-Symmetrically substituted systems: 1,1′-dibismuthanylferrocenes*		
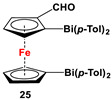	(*α*-CHO-C_5_H_4_)(C_5_H_5_)Fe, Me_2_N(CH_2_)_2_NMeLi, and *^n^*BuLi, and (*p*-Tol)_2_BiCl in the next step [111].	No application reported for **25** [111].
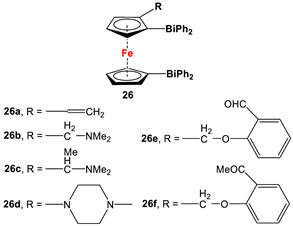	(*α*-CH_2_NMe_2_-C_5_H_3_)(C_5_H_5_)Fe, *^n^*BuLi, and Ph_2_BiCl (Scheme 3D) for **26b**, [112] which acted as a precursor for other species.	No application reported for **26** [112].
*1,1′-Unsymmetrically substituted systems: 1,1′-aminophosphanylferrocenes*		
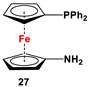	Fc’(PPh_2_·BH_3_)N_3_ and DABCO (Scheme 4A) [113].	No application reported for **27** [113].
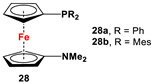	Fc’(NMe_2_)Br, *^n^*BuLi, and R_2_PCl (R = Ph and Mes) [114].	Isolated complexes with compound **28a** were reported with PdCl_2_, PdCl(SbF_6_), PPh_3_(BF_4_)_2_, PdPPh_2_Cp(SbF_6_)_2_, [**28a**·Pd(PPh_2_)Fc’(NMe_2_)], and P(*p*-OMe-C_6_H_4_)_3_(BF_4_)_2_ [114].
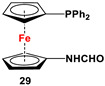	Fc’(PPh_2_·S)N_3_, Raney Ni, and HCOOAc (Scheme 4B) [113].	Compound **29** was used to synthesize **32a** [113].
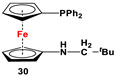	**30**·S and Raney Ni [115].	No application reported for **30** [115].
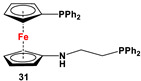	Fc’(PPh_2_·BH_3_)NH_2_ and Ph_2_PCH_2_CO_2_H·BH_3_, followed by DABCO [116].	Isolated complexes with compounds **31** are reported with PdCl_2_, PdCl(SbF_6_), and PdCl(SbF_6_)_2_ [116].
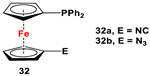	**29** and BOP/DBU for **32a** [113], Fc’(Ph_2_P·BH_3_)Br or Fc’(Ph_2_P·S)Br, and *^n^*BuLi and TsN_3_ for **32b**, followed by removal of BH_3_ or S [113].	Isolated complexes with compound **32a** are reported with AgCl [113], Ag(SbF_6_) [113], Ag(Me_2_CO)(SbF_6_) [113], AuCl [113], (AuCl)_2_ [113], (AuCN)_2_(SbF_6_)_2_ [113], and (AuCN)_2_(NTf)_2_ [113]. Au-complexes of **32a** were used for cyclodimerization of enynol [113]. Fischer-type and Mesoionic carbenes were also synthesized from **32a** and **32b**, respectively [117,118].
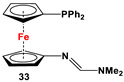	Fc’(PPh_2_)NH_2_ and Me_2_NCH(OMe)_2_ [119].	Isolated complexes with compounds **33** are reported with PdCl_2_, PdCl(BARF) (BARF = B(3,5-(CF_3_)_2_C_6_H_3_)_4_), (*η*^6–^1-Me, 3-*^i^*Pr-C_6_H_4_)Ru, and (*η*^6^-C_5_Me_5_)Rh [119].
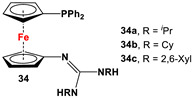	Fc’(PPh_2_)NH_2_, *^n^*BuLi, and respective RNCNR [120,121].	Isolated complexes with compounds **34** are reported with PdCl_2_ [120], PdCl(SbF_6_) [120], PdCl(BF_4_) [120], PtCl_2_ [121], PdCl(BARF) [121], PdBr(4-CN-C_6_H_4_) [121], and Pd(4-CN-C_6_H_4_)SbF_6_ [121]. Isolated complexes with [**34**·H]SbF_6_ were reported with PdCl_2_ and *μ*-Pd_2_Cl_4_ [120].
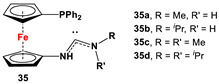	**35**·PdCl_2_: Fc’(PPh_2_)NC, PdCl_2_(cod), and respective RR’NH [118].	Isolated complexes with compound **35** are reported with PdCl_2_, which was further reported with Miyaura borylation [118].
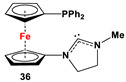	**36**·PdCl_2_: Fc’(PPh_2_)NC, PdCl_2_(cod), and respective [Cl(CH_2_)_2_NH_2_Me]Cl [118].	Isolated complexes with compound **36** are reported with PdCl_2_, which was further reported with Miyaura borylation [118].
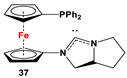	**37**·PdCl_2_: Fc’(PPh_2_)NC, PdCl_2_(cod), and (*S*)-2-(chloromethyl)pyrrolidine [118].	No application reported for **37** [118].
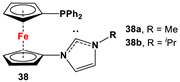	**38**·PdCl_2_: Fc’(PPh_2_)NC, PdCl_2_(cod), and respective (MeO)_2_CHCH_2_NHR (R = Me, *^i^*Pr) [118].	Isolated complexes with compound **38** are reported with PdCl_2_, which was further reported with Miyaura borylation [118].
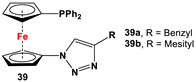	Fc’(PPh_2_·BH_3_)N_3_, RC≡CH (R = Bz, Mes), and CuSO_4_·5H_2_O [122].	Isolated complexes with compound **39** are reported with MeBF_4_, PdCl_2_(MeBF_4_), Au*^i^*Pr(MeBF_4_), and [PdCl_2_(Au*^i^*Pr)_2_(MeBF_4_)_2_]_1/2_ [122].
*1,1’-Unsymmetrically substituted systems: 1,1’-arsanylphosphanylferrocenes*		
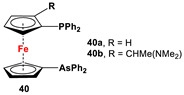	[1]PhosphaFCP and *^n^*BuLi, followed by Ph_2_AsCl [123,124].	No application reported for **37** [123,124,125].
*1,1′-Unsymmetrically substituted systems: 1,1′-phosphanylstibanylferrocene*		
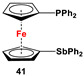	Fc’(PPh_2_)Br and *^n^*BuLi, followed by Ph_2_SbCl [126].	Isolated complexes with compound **41** are reported with AuCl, which was further treated with 3,5-di-tert-butyl-o-benzoquinone for further complexation with Sb. Both complexes were eventually used for gold catalysis [127].

**Scheme 2:**
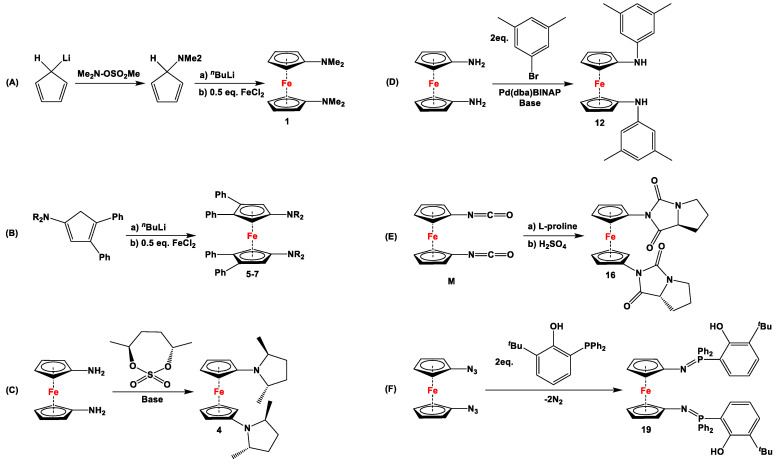
Synthetic routes to selected 1,1′-N,N-ferrocenes (**A**–**F**) [31,49,55,56,58,99].

**Scheme 3:**
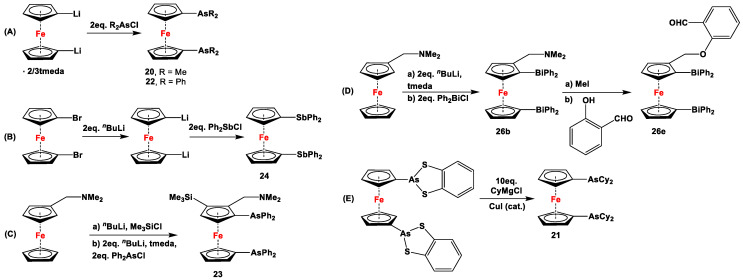
Synthetic routes to 1,1′-diarsanyl- (**A**,**C**,**E**), 1,1′-distibanyl- (**B**), and 1,1′-dibismuthanylferrocenes (**D**) [2,109,110].

**Scheme 4:**
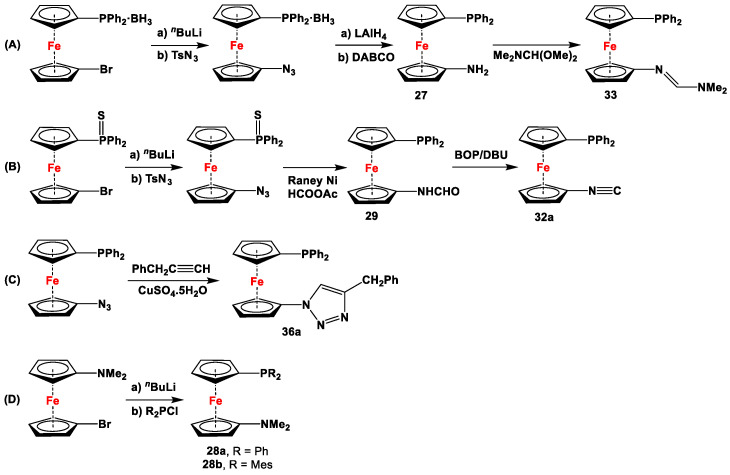
Synthetic routes for 1,1′-N,P-ferrocenes (**A**–**D**), where DABCO, BOP, and DBU stand for 1,4-diazabicyclo [2.2.2]octane, (benzotriazol-1-yloxy)tris(dimethylamino)phosphonium hexafluorophosphate, and 1,8-diazabicyclo(5.4.0)undec-7-ene, respectively [113,114,119,122].

## 3. Synthetic Aspects

The earliest example of 1,1′-symmetrically substituted diaminoferrocene was synthesized via a “modified fly-trap methodology”, where a general entry of cyclopentadienylamine is provided by the reaction of C_5_H_5_Li with the hydroxylamine derivative of Me_2_N-OSO_2_Me. The resulting C_5_H_5_NMe_2_ was then deprotonated and in situ reacted with FeCl_2_ to obtain title compound **1** (Scheme 2A) [49]. This methodology has further been extended to synthesize 3,4,3′,4′-tetraphenyl-substituted 1,1′-diaminoferrocene (**5**–**7**), where a family of 3,4-diphenyl-substituted cyclopentadienylamine has initially been used for deprotonation and subsequent salt metathesis reactions with FeCl_2_ (Scheme 2B) [57,58,59,60]. While the above-mentioned “modified fly-trap methodology” is restricted to Fc′(NMe_2_)_2_ (**1**) and a few Ph_4_-substituted 1,1′-diaminoferrocenes **5**–**7** [57,58,59,60], the majority of other diamines were typically synthesized using a series of well-established synthetic methodologies, starting from Fc′(NH_2_)_2_ (selected examples in Scheme 2C,D) [55,56,61,81,91], which is synthesized either via catalytic hydrogenation of Fc′(N_3_)_2_ [128] or via two-step Gabriel-type synthesis, starting from Fc’Br_2_ or Fc’I_2_ [35]. Other case-specific synthetic strategies have occasionally been employed to access **16** and **19**, involving condensation and Staudinger reactions, starting from **M** and Fc′(N_3_)_2_, respectively (Scheme 2E,F) [31,99].

Dimethyl- and diphenyl-substituted 1,1′-diarsanyl- and 1,1′-distibanylferrocenes **20**, **22**, and **24** were synthesized by salt metathesis reactions of tmeda-stabilized (i.e., Fc’Li_2_·2/3tmeda) [129] or in situ-synthesized Fc’Li_2_ with Me_2_AsCl (Scheme 3A) [2], Ph_2_AsCl (Scheme 3A) [2], and Ph_2_SbCl (Scheme 3B) [110], respectively. A similar methodology has further been applied for plana r-enantiomeric versions of 1,1′-distibanyl- and dibismuthanylferrocenes **23** and **26b**, where -CH_2_NMe_2_ units guided the corresponding lithiation to *α*-Cp positions (Scheme 3C,D) [109,112,130,131,132]. Compound **26b** was further treated with a series of common organic reagents, giving rise to a family of 1,1′-dibismuthanylferrocenes with different pendant difunctional substituents (selected example of **26e** in Scheme 3D) [112]. On the other hand, 1,1′-bis(dicyclohexylarsanyl)ferrocene (**21**) was synthesized via a CuI-catalyzed reaction of 1,1′-bis(dithiaarsole)ferrocene with an excess amount of cyclohexylmagnesium chloride (Scheme 3E) [106].

The main precursors for all previously reported N,P-substituted ferrocene ligands (such as **27** and Fc′(PPh_2_)N_3_) have been synthesized, starting from protected ferrocenyl phosphanes to avoid unwanted Staudinger reactions. For example, when Fc′(Ph_2_PBH_3_)Br was selectively lithiated and subsequently reacted with TsN_3_, Fc′(Ph_2_PBH_3_)N_3_ was obtained. In the next step, Fc′(Ph_2_PBH_3_)N_3_ was further reduced and deprotected to obtain title compound **27** (Scheme 4A) [113]. Alternatively, thionation has been used to protect the P-functionality. To this end, selective lithiation was first performed on Fc′(Ph_2_P = S)Br, followed by salt metathesis reaction with TsN_3_. The resulting Fc′(Ph_2_P = S)N_3_ was either reduced selectively at the P-functionality to obtain Fc′(PPh_2_)N_3_ (Scheme 4A), or the P and N-functionalities simultaneously transformed to title compound **32a** (Scheme 4B) [113]. Starting from **27** and Fc′(PPh_2_)N_3_, a family of 1,1′-azaphospha ferrocenylene ligands (such as **29** [113], **30** [115], **31** [116], **32a** [113], **33** [119], **35** [118], and **36** [122], has been accessible using a series of well-established synthetic methodologies, as outlined in Scheme 4A,C. On the other hand, the syntheses of ligands **28** was based upon the successful and large-scale preparation of an unsymmetrically substituted 1,1′-aminobromoferrocene Fc′(NMe_2_)Br (Scheme 4D), which was first lithiated and subsequently reacted with R_2_PCl (where R = Ph and Mes) to obtain target compounds **28a** and **28b** [114]. Here, it is to be noted that in order to synthesize **28**, N (i.e., NMe_2_) was first introduced at ferrocene, followed by P (i.e., PR_2_), whereas an opposite synthetic order was followed for Fc′(PPh_2_)N_3_, **27**, and **32a** (compare Scheme 4A,B,D) [113].

**Scheme 5:**
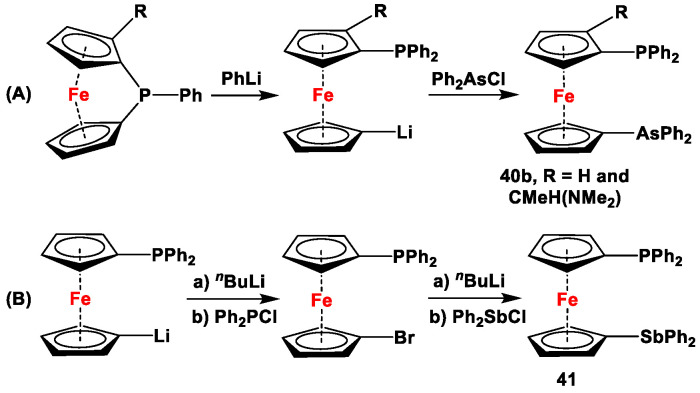
Synthetic routes for 1,1′-arsanylphosphanyl- (**A**) and 1,1′-arsanylstibanylferrocenes (**B**) [113,114,119,122,123,124,125,126].

1,1′-Arsanylphosphanylferrocenes have been synthesized via ring-opening reactions of phospha [1]FCPs, where PhLi has reportedly been used as a ring-opening agent. The resulting anionic species were further in situ reacted with Ph_2_AsCl to selectively synthesize **40b** (Scheme 5A) [123,124,125]. On the other hand, the only example of 1,1′-phosphanylstibanylferrocene was synthesized in a modular approach, where 1,1′-dibromoferrocene was first selectively lithiated and subsequently reacted with Ph_2_PCl. The resulting Fc′(PPh_2_)Br was further lithiated and in situ reacted with Ph_2_AsCl to synthesize mixed compound **41** (Scheme 5B) [126].

## 4. Complexation Motifs of Pnictogen-Substituted 1,1′-Ferrocenes

**Figure 2:**
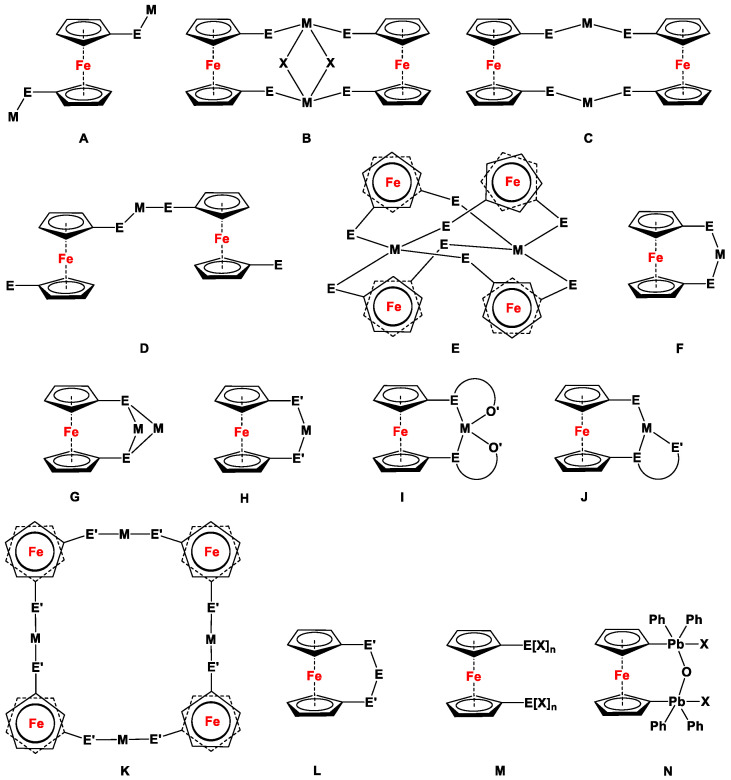
Simplified illustrations demonstrating the binding modes for pnictogen-disubstituted 1,1′-ferrocenes: open- (**A**), quasi-closed- (**B**), and double-bridged complex (**C**); lower (**D**) and higher aggregated *η*^1^, *η*^1^-interconnected complex (**E**); chelated (**F**) and double-chelated complex (**G**); cyclic ((**H**), E’ = FcNR^−^) and multidentate chelated species ((**I**,**J**), O’ = PhO^−^, E’ = FcNR^−^); higher-aggregated species with transition metal bridges ((**K**), E’ = PhO^−^); tetrylene-bridged species ((**L**), E = carbene, silylene, stannylene, and germylene), compounds obtained via oxidation of **24** ((**M**), where X = F and Cl, n = 2 and 4; and (**N**), where X = F, Cl, ONO_2_, and OClO_3_). Note For the sake of simplicity, top views of the complexes are depicted for (**E,K**).

The steric situation of the phosphanyl units in dppf analogs is one of the key features of these compounds, allowing for a wide variation of complexation modes [7]. By contrast, the pnictogen-substituted non-phosphanyl species show only a handful examples for open-bridged (entries 1–10, Table S1, ESI; Figure 2A), quasi-closed-bridged (entry 11, Table S1, ESI; Figure 2B), double-bridged (entries 12 and 13, Table S1, ESI; Figure 2C), lower- (entries 14–16, Table S1, ESI; Figure 2D), and higher-order *η*^1^, *η*^1^-interbridged (entry 17, Table S1, ESI; Figure 2E) complexes. However, 1,1′-bisimino- (**17a** and **17i**), diarsanyl- (**20** and **22**), distibanyl- (**24**), and phosphanyliminoferrocenes (**33** and **34a**) predominantly show chelation as their preferred mode of complexation (entries 23–54; Figure 2F), which are further compared with similar complexes from dppf analogs (entries 18–22), and a rare example of double chelation for **17j** (Entry 55; Figure 2G) in Table S1 (ESI). When secondary amines (**9**–**11**) and substituted imines with proximal hydroxyl groups (**18** and **19**) were deprotonated and in situ reacted with metal halides, cyclic (entries 56 and 57, Table S1, ESI; Figure 2H), multidentate chelated (entries 58–81, Table S1, ESI; Figure 2I,J), and higher-order species with intermolecular N-M-N bridges (entries 82 and 83, Table S1, ESI; Figure 2K) were obtained. 1,1′-Diaminoferrocenes with secondary amines (**9** and **11**) have notably been found to be useful in stabilizing carbenes, silylenes, germylenes, and stannylenes, which are further showcased as entries 84–120 in Table S1 (ESI) (Figure 2L). In order to present a complete picture to the readers, Table S1 (ESI) has further been equipped with compounds (entries 121–127; Figure 2M,N), obtained by oxidation of 1,1′-distibanylferrocene **24**, featuring a rare family of SbOSb [3]FCPs (entries 124–127; Figure 2N).

Each complexation mode is exemplified with examples, which are arranged following the order of Table 1. Complexes from each ligand were then arranged by increasing atomic number of the corresponding complexation partners in Table S1 (ESI). As the complexes with deprotonated ligands (entries 58–81, Table S1, ESI) exhibit E-M distances in an acceptable range of polar coordination bonds and tetrylene-bridged cyclic species (entries 84–120, Table S1, ESI) structurally behave similar to [3]FCPs, the corresponding dihedral angles (*α*) are recorded for them in Table S1 (ESI). In order to complete the data-set, dihedral (*α*) angles are listed for all chelating complexes and cyclic species, in contrast to open- (entries 1–10, Table S1, ESI; Figure 2A) and *η*^1^, *η*^1^-interconnecting complexes (entries 14–16, Table S1, ESI; Figure 2D,E). No meaningful bite angles (*β_n_*) have been defined for open- (entries 1–10; Figure 2A), quasi- (entry 11; Figure 2B), double-bridged (entries 12 and 13; Figure 2C), lower- (entries 14–16; Figure 2D), or higher-order *η*^1^, *η*^1^-interconnected complexes (entry 17; Figure 2E) in Table S1, ESI. For acyclic coordination such as for **24**(F)_2_, **24**(F)_4_, and **24**(Cl)_4_ (entries 121–123; Figure 2M), and higher-order compounds with intermolecular N-M-N bridges (entries 82 and 83, Table S1, ESI; Figure 2N), *α* and *β_n_* are not listed. Similarly, Fischer-type carbenes are not included in this table, as they do not feature any direct bonding connectivity between transition metal and a donor atom directly attached to the ferrocene.

**Figure 3:**
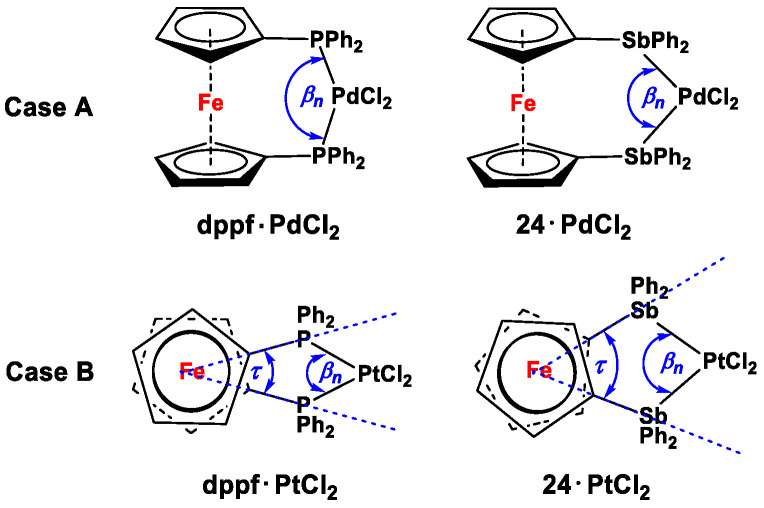
Simplified illustrations for the dependence of *β_n_* on the lengths of E-M bonds (case (**A**)), Sb-Pd > P-Pd, *β_n_*^dppf·PdCl2^ > *β_n_***^24^**^·PdCl2^) and Cp/Cp twist angles *τ* (case (**B**)), *τ* **^24^**^·PdCl2^ > *τ* ^dppf·PdCl2^, *β_n_***^24^**^·PdCl2^ > *β_n_*^dppf·PdCl2^). Note Dihedral angle *α* is not being considered while drawing for case (**A**), and the Cp rings were consequently drawn as parallel to each other.

After methodically arranging the entire wealth of complexes and compounds derived from ligands **1**–**41**, our next aim was to compare bite angles (*β_n_*) for bisphosphanyl and bisarsanyl ligands. In order to do so, Pd(II) complexes of dppf and its analogs (dppf·Pd(*η*^2^-C_60_), dppf·PdCl_2_, and Fc′(PMes_2_)(P*^t^*Bu_2_) PdCl_2_ in entries 18, 19, and 22, respectively, in Table S1, ESI) were first structurally compared with **24**·PdCl_2_ (entry 39, Table S1, ESI), where the *β_n_*s (complex type Figure 2F) were found following the trend of dppf·Pd(*η*^2^-C_60_), dppf·PdCl_2_, and Fc′(PMes_2_)(P*^t^*Bu_2_)·PdCl_2_ > **24**·PdCl_2_. This trend possibly resulted from the long Sb-Pd bonds (2.5020(5) Å), which push the bridging PdCl_2_ moiety away from the ferrocenylene unit, decreasing the *β_n_* compared to dppf and its analogs (where P-Pd = 2.262(4)-2.286(3) Å; case Figure 3A). This argument holds true for a similar comparison between dppf·Pt(*η*^2^-C_60_) and **24**·PtCl_2_ (entries 20 and 43, Table S1, ESI), where the latter showed higher *β_n_* compared to the former. However, on comparison between **24**·PtCl_2_ and dppf·PtCl_2_ (entries 43 and 21, Table S1, ESI), an opposite trend could also be found, where, despite longer Sb-Pt bonds (2.5007(5) Å) and a smaller *α* angle (1.0°), the former complex showed a higher value of *β_n_* (96.49(1)°, complex type Figure 2F) than the latter (P-Pt = 2.266(5) Å, *α* = 5.0°, and *β_n_* = 91.6(2)°). However, a close inspection of their structural features reveals larger twist angle in **24**·PtCl_2_ (32.6°), making *β_n_* higher than that of dppf·PtCl_2_ (tilt angle 30.7°, case Figure 3B).

When comparing complexes of a ligand scaffold with different substituents, bite angles (*β_n_*) increase with enhanced steric interactions. For example, *β_n_*s for complexes of **17a**, **24**, and **34a** showed the following trends: **17a**·PdClMe > **17a**·PdCl_2_ (entries 23 and 24, Table S1, ESI), **24**·[Ru(*η*^6^-1-Me,3-*^i^*Pr-C_6_H_4_)Cl][PF_6_] > **24**·[Ru(*η*^5^-C_5_Me_5_)Cl] (entries 36 and 37, Table S1, ESI), and **34a**·PdBr(*p*-CN-C_6_H_4_) > [**34a**·Pd(acac)](SbF_6_) (entries 49 and 52, Table S1, ESI). On the other hand, when the PdCl_2_ complex of sterically bulky ligand **17i** was compared with that of its slimmer counterpart **17a**, *β_n_*s showed the expected trend, i.e., **17i**·PdCl_2_ > **17a**·PdCl_2_ (entries 23 and 32, Table S1, ESI). Chelate complexes, such as **24**·PdCl_2_ (entry 39, Table S1, ESI), showed a larger *β_n_* value than their counterparts with a shared metal cation, such as (**24**)_2_·(*μ*-Pd)(SbF_6_) (entry 40, Table S1, ESI). This is likely due to the steric interactions between two adjacent ligand molecules of **24**, which is further supported by the elongation of the Sb-Pd distances from **24**·PdCl_2_ (2.5020(5) Å) to (**24**)_2_·(*μ*-Pd)(SbF_6_) (2.6142(4) Å). Despite having a pool of complexes with different metal ions, intraspecific comparisons did not deliver any clear trends for complexes with **17i** (entries 25–32, Table S1, ESI) and **24** (entries 36–45, Table S1, ESI).

Considering the effect of secondary ligands, Zr-complexes of **11d** feature higher steric congestion in (**11d**-2H)Zr(CH_2_Ph)_2_ than in (**11d**-2H)Zr(NMe_2_)_2_, based on the larger *β_n_* angle (complex type **H**, Figure 2) in the former (112.94(11) Å, entry 57, Table S1, ESI) than in the latter (104.68(15) Å, entry 56, Table S1, ESI). Similarly, by comparison of multidentate chelated species from **18e**, (**18e**-2H)Zr(O*^i^*Pr)_2_ (*β_n_* = 98.26°, entry 72, Table S1, ESI) showed higher *β_n_* (complex type **I**, Figure 2) than that of (**18e**-2H)Zr(O*^n^*Pr)_2_ (*β_n_* = 96.93°, Entry 71, Table S1, ESI). However, despite increased steric bulk and decreased *α* angle, *β_n_* surprisingly decreased on moving from (**18e**-2H)Zr(O*^i^*Pr)_2_ (*α* = 5.2°, *β_n_* = 98.26°, entry 72, Table S1, ESI) to (**18e**-2H)Zr(O*^t^*Bu)_2_ (*α* = 3.1°, *β_n_* = 85.90°, entry 73, Table S1, ESI), which is speculatively due to elongation of O^Ph^-Zr distance in the latter (from 2.031 Å to 2.120(1) Å for (**18e**-2H)Zr(O*^i^*Pr)_2_ and (**18e**-2H)Zr(O*^t^*Bu)_2_, respectively).

Tetrylene-bridged 1,1′-diamino-ferrocenes exhibit an easily comprehendible relation between dihedral angles (*α*) and corresponding bridging elements, where, *α* varies in a substantially wider range for carba- (*α* = 15.4–18.3°), sila- (*α* = 6.2–16.4°), germa- (*α* = 5.5–10.2°), and stanna-bridged compounds (*α* = 1.9–5.6°), listed in entries 84–120 (Table S1, ESI). Similar to [*n*]FCPs, the dihedral angles (*α*) increase and decrease with the size of the bridging element. For example, when tetrylene-bridged species derived from **9** and **11** were compared, the following trends were observed for *α* angles: (**9a**-2H)Ge, (**9a**-2H)Ge(SePh)_2_ > (**9a**-2H)Sn (entries 84–86, Table S1, ESI); (**9b**-2H)C, [(**9b**-2H)CH][BF_4_], (**9b**-2H)[C-RhCl(cod)] > (**9b**-2H)Ge > (**9b**-2H)_2_Sn (entries 87–91, Table S1, ESI); [(**9d**-2H)CH][BF_4_], (**9d**-2H)C > (**9d**-2H)Ge, [(**9d**-2H)Ge(*μ*-S)]_2,_ [(**9d**-2H)Ge(*μ*-Se)]_2_, (**9d**-2H)(Ge_3_OCl_2_) (entries 92–97, Table S1, ESI); (**9e**-2H)Ge, (**9e**-2H)Ge(SePh)_2_ > (**9e**-2H)Sn (entries 98–100, Table S1, ESI); (**11b**-2H)Si(SePh)_2_, (**11b**-2H)Ge, (**11b**-2H)Ge(SePh)_2_, [(**11b**-2H)Ge(*μ*-Se)]_2_, [(**11b**-2H)Ge]_2_·(*μ*-Mo(CO)_4_) > (**11b**-2H)Sn (entries 104–109, Table S1, ESI); (**11c**-2H)Si > (**11c**-2H)Ge (entries 110 and 118, Table S1, ESI); and (**11c**-2H)Si(SePh)_2_ > (**11c**-2H)Ge(SePh)_2_·1/2C_6_H_6_ (entries 113 and 120, Table S1, ESI), where “≈” (almost equal to) and “>” (greater than) were used to indicate trends for a given parameter (i.e., *α*).

**Figure 4:**
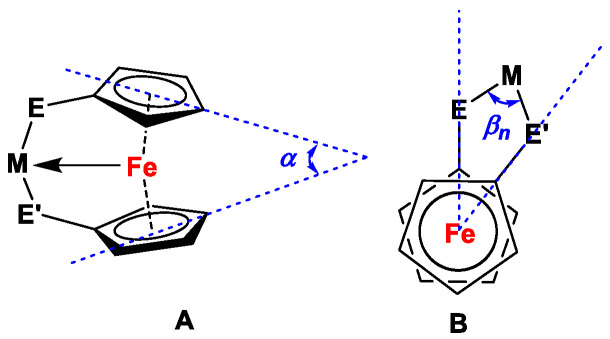
Molecular parameters of Pd(II)/Ni(II) complexes of dppf analogs with Fe→Pd/Ni interactions with tilt (*α*, (**A**)) and bite (*β_n_*, (**B**)) angles.

In the next step, the chelated complexes with Fe→Pd and Fe→Ni interactions are discussed, and Table S2 (ESI) summarizes all related species for ligands **17**, **28**, **31**, and **34**. Their molecular parameters (such as avg. C*^ipso^*^,Cp^-E bond lengths, Ni/Pd-Fe distances, and tilt and bite angles) will further be compared with the corresponding values for similar complexes with P,P-substituted dppf analogs (entries 1–3, 26, and 27, Table S2, ESI). When analyzing and discussing the lengths of Fe-Pd distances for cationic Pd(II)-complexes with Fe-Pd interactions, it is observed that the differences in C*^ipso^*^,Cp^-P or C*^ipso^*^,Cp^-N bond lengths majorly affect the respective Pd-Fe distances and interactions for related P,P-, N,N-, or P,N-analogs of dppf. Shorter Pd-Fe distances are observed for ferrocene-based N,N ligand scaffolds (Pd-Fe = 2.6297(4)-2.7954(5) Å for complexes with **17c** and **17e**–**17i**; entries 4–16, Table S2, ESI) compared with their P,P counterparts (Pd-Fe = 2.7974(10)-3.0014(4) Å for complexes with dppf and Fc’(PMes_2_)(P*^t^*Bu_2_); entries 1–3, Table S2, ESI), whereas for related mixed P,N scaffolds (Pd-Fe = 2.7384(18)-2.8349(11) Å for complexes with **28a**, **31**, **34a**, **34b**, and **34c**; entries 17–25, Table S2, ESI), intermediate Pd-Fe distances can be seen. Similar trends could also be observed for complexes with Fe-Ni interactions, where complexes with N,N-substituted dppf analogs show shorter Ni-Fe distances (Ni-Fe = 2.6268(4)-2.8244(6) Å for complexes with **17e** and **17i**; entries 28–30, Table S2, ESI) than those of corresponding P,P-substituted counterparts (Ni-Fe = 3.498 Å for complexes with (C_5_H_4_P*^i^*Pr_2_)Fe; entries 26 and 27, Table S2, ESI). However, the weakest Fe→M interactions and, consequently, the longest Fe-M bond distances could be observed for Sc (III), Y (III), La (III), and Lu (III) compounds, listed in entries 31–34 (Table S2, ESI), where, despite having short C*^ipso^*^,Cp^-E bond lengths (1.366(8)-1.401(7) Å), the Fe-M bond distance varies between 3.158(2) and 3.3857(8) Å. It is also to be noted that ferrocene moieties in these complexes are tilted in the opposite direction of the E-Pd-E’ or E-Ni-E’ bridges, and the Pd-Fe or Ni-Fe distances are not a consequence of either steric repulsion or any sort of geometric distortions alone in the related molecules (Figure 4). As per Pietschnig and co-workers, intermetallic distances in these complexes result from a compromise between minimized steric repulsions, rotational distortions, and secondary interactions of the ligand systems in the solid state [114].

**Figure 5:**
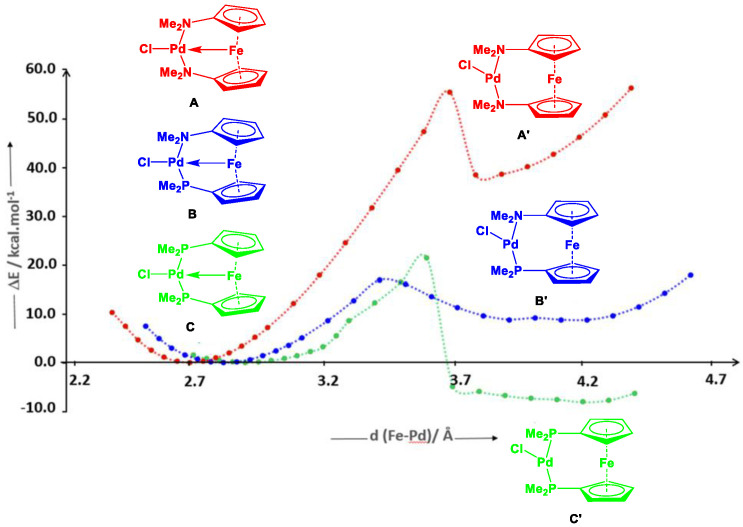
Potential energy surface scan for the elongation of the Fe-Pd distances in the model compounds containing (N,N’-), (N,P-), and (P,P’-) donating centers. Reproduced from Ref. [114] with permission from the Chinese Chemical Society (CCS), Peking University (PKU), and the Royal Society of Chemistry.

DFT calculations performed on these complexes were further able to verify their intrinsic structural features and trends, which further shed light on the nature of the Fe→Pd bonding interactions. In case of [**17i**·Pd(NCMe)][BF_4_]_2_ and [**17i**·NiPh][BPh_4_] (entries 16 and 28, Table S2, ESI), Tamm and co-workers demonstrated the possible existence of second minima on the potential energy surface, where the Pd-Fe distance is significantly longer [87,133]. Eventually, Pietschnig and coworkers further increased the distances between the Pd and Fe centers in case of model systems **A**–**C**, where second minima were found around ~3.78 Å (**A’**), ~4.01 Å (**B’**), and ~4.29 Å (**C’**), and the Pd atom adopted a slightly distorted T-shaped geometry, which in turn complies with earlier knowledge (Figure 5) [133]. In contrast to **A** and **B**, in the case of **C**, the T-shaped second minima (**C’**) were more stable by 8.1 kcal mol^−1^ (ΔE_isomer-scan_, Figure 5). Pietschnig and co-workers came to an additional conclusion, where the introduction of bulky substituents at the donor atoms prevent the formation of the T-shaped isomer, which can further prevent dimerization via the formation of an intermolecular Pd_2_Cl_2_ bridging unit. Therefore, once the chlorine substituent on Pd was replaced with more bulky phosphanes in [**28a**·Pd(PPh_2_C_5_H_5_)][SbF_6_]_2_, [**28a**·Pd(PPh_3_)][BF_4_]_2_, [**28a**·Pd(PPh_2_)Fc′(NMe_2_)][BF_4_]_2_, and [**28a**·PdP(*p*-OMe-C_6_H_4_)_3_][BF_4_]_2_, due to steric factors, the stability of the T-shaped molecular geometry at Pd centers substantially decreased compared to [**28a**·PdCl][SbF_6_]_2_ [114].

## 5. Electronic Situation in Pnictogen-Disubstituted 1,1′-Ferrocenes and Their Complexes

The electrochemistry of ferrocenyl amines have been studied extensively for decades. As per Britton and Herberhold et al., the correlation of oxidation potentials for ferrocenyl moieties are in good agreement with Taft’s constants (σ^o^_p_) rather than Hammet’s constants (σ), which indicates predominant resonance effects of N atoms over to their inductive effects [51]. Such efficient N-to-Cp electron donations result in electron-rich Fe centers, which show considerably low redox potentials for 1,1′-N,N-substituted species (such as **1**, **2**, **4**–**7**, **9b**–**9e**, **11b**, and **19**; entries 5–16, 19, and 20, Table S3, ESI) and 1,1′-P,N-substituted species (**33** and **34a**; entries 26 and 27, Table S3, ESI), with respect to those of ferrocene and dppf (entries 1 and 2, Table S3, ESI). N-to-Cp extended electronic conjugations can further be supported by shortening N-C^Cp^ bonds (1.377(2) Å for **4**) and planar N atoms [56]. For 1,1′-diiminoferrocenes with CHAr substituents (**17a** and **18e**), despite having planar N centers (N-C^Cp^ = 1.397(4) Å for **18b**, isostructural with **18e**) [89], their lone pair is partially conjugated with phenyl groups, which further decreases N-to-Cp electron donation and consequently increases the values of E^0^ (entries 17 and 18, Table S3, ESI). On the other hand, due to featuring non-planar N atoms, N-to-Cp electron donations are not fully supported for **28a** and **28b**, resulting in a substantial increase in E^0^ (entries 23 and 24, Table S3, ESI). Owing to a substantial energy difference between 2p and 5p orbitals, the lone pairs of Sb are not conjugated with the Cp rings, resulting in tetrahedral Sb moieties and relatively high E^O^ values (entry 21, Table S3, ESI). It is here noteworthy that Fe→Pd interactions in **34a**·PdCl(SbF_6_) and [**28a**·Pd(PPh_2_)Fc′(NMe_2_)][BF_4_]_2_ are fairly strong and, consequently, the Fe atom is sparingly available for reversible oxidation (entries 36 and 46, Table S3, ESI).

As the coordination complexes are formed by donating lone pairs of electrons from N to corresponding metal ions, N-to-Cp electron donations become no longer possible, and as a consequence of such restricted conjugation, their E^0^ values increase from **5** to **5**·Zn(CF_3_SO_3_)_2_, **5**·[Zn(CF_3_SO_3_)_2_]_2_, **5**^Chelate^·Zn(CF_3_SO_3_)_2_, **5**·Co(CF_3_SO_3_)_2_, **5**·[Co(CF_3_SO_3_)_2_]_2_, and **5**^Chelate^·Co(CF_3_SO_3_)_2_ (entries 9, 28–33, Table S3, ESI); from **17a** to **17a**·PdMeCl and [**17a**·PdMeCl]BAF (entries 17, 35 and 36, Table S3, ESI); from **28a** to [**28a**^chelate^]**28a**·Pd(BF_4_)_2_ (entries 23 and 40, Table S3, ESI); from **32a** to **32a**·AuCl, [**32a**·AuCl]_2_, and [**32a**·*μ*-Au]_2_X_2_ (X = SbF_6_, NTf_2_) (entries 25, 41–43, Table S3, ESI); from **33** to **33**·PdCl_2_ (entries 26 and 44, Table S3, ESI); and from **34a** to **34a**·PdCl_2_, **34a**·PdCl(SbF_6_), and **34a**·PdCl(SbF_6_) (entries 27, 45, and 46, Table S3, ESI). Although P and Sb centers in dppf and **24** do not have lone pairs suitable for P/Sb-to-Cp donations, a negative inductive effect has been considered upon complexation for dppf·PdCl_2_, Fc′(PMes_2_)(P*^t^*Bu_2_)·PdCl_2_, **24**·PdCl_2_, and **24**·[Pd(*η*^2^-maleic anhydride)], accompanied by increasing E^0^ values for dppf and **24** versus their respective complexes (entries 3, 4, 37, and 38, Table S3, ESI). For compounds with covalently bonded metal bridges and tetrylenes, the increase in E^0^ values is accompanied by the formation of N-E (where E = metal atoms and tetrylenes) bonds, forcing the N atoms from planar configuration to tetrahedral, and restricting N-to-Cp extended electronic conjugations. For example, E^0^ values increased from **9b** to [**9b**-2H]CH[BF_4_], [**9b**-2H]C, [**9b**-2H][C-RhCl(CO)_2_], and [**9b**-2H]Ge (entries 12, 50–53, Table S3, ESI); from **9c** to [**9c**-2H]Ge (entries 13 and 54, Table S3, ESI); from **9d** to [**9d**-2H]C (entries 14 and 55, Table S3, ESI; from **9e** to [**9e**-2H]Ge (entries 15 and 56, Table S3, ESI); from **11b** to [**11b**-2H]Ge (entries 16 and 57, Table S3, ESI); from **18e** to (**18e**-2H)AlO*^i^*Pr, (**18e**-2H)Zn, (**18e**-2H)Co, (**18e**-2H)Y(O*^t^*Bu)THF, (**18e**-2H)Ce(O*^t^*Bu)_2_, and (**18e**-2H)Ce(O*^t^*Bu)THF (entries 18, 61–66, Table S3, ESI); from **19a** to (**19a**-2H)Y(O*^t^*Bu), (**19a**-2H)Ce(O*^t^*Bu)THF, and (**19a**-2H)Ce(O*^t^*Bu)_2_ (entries 19, 67–69, Table S3, ESI); and from **19b** to (**19b**-2H)YCl and (**19b**-2H)Y(CH_2_Ph) (entries 20, 70, and 71, Table S3, ESI).

## 6. Applications

The multitude of applied aspects has been sorted into two major divisions: redox-active sensoric materials, where no catalytic reactivity is involved, and catalytic reactions, where the respective ligands have first been used to synthesize isolable or in situ prepared metal complexes, which have been used for various catalytic reactions. On the basis of applications, the non-catalytic reactions were further classified into the following sub-topics: redox-responsive molecular switches, ion recognition receptors, mesoionic and Fischer-type carbenes, dearomatization reactions of N-heterocycles, and the exploration of oxidation reactions on germylenes. On the other hand, the catalytic reactions are categorized under the following sub-headings: ring-opening polymerization of lactides and cyclic esters, Pd-catalyzed cross-coupling reactions, and Au-catalyzed annellation reactions.

### 6.1. Redox-Active Sensoric Materials

#### 6.1.1. Redox-Responsive Molecular Switches

**Scheme 6:**
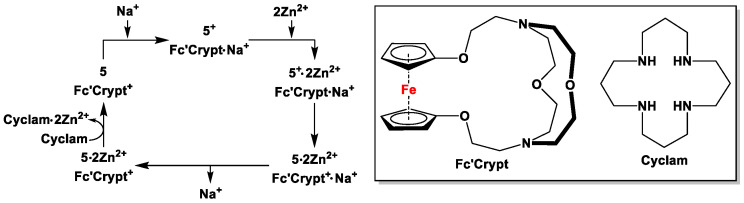
Electron-transfer-mediated regulation of the Na^+^ concentration by Zn^2+^ ions [59,60].

The most interesting aspect for non-catalytic applications is that of molecular switches, where a characteristic property of a molecule can reversibly be switched on or off by changing the oxidation state of the organometallic scaffold, which is coupled to a macrocyclic ligand. In molecular switches, the coordination of a metal cation is destabilized upon oxidation of the redox-active unit, and can further be restabilized upon reduction of the same. The concept of molecular switches on N-containing dppf analogs were first introduced by Plenio et al. [59], where a molecular switch was coupled with a redox-responsive ligand **5**. The interaction of a redox-responsive chelating aminoferrocene **5**, a redox-switchable oxaferrocene cryptand (**Fc**′**Crypt**), with Zn^2+^ and Na^+^ is shown in Scheme 6. The addition of two equivalents of Zn(CF_3_SO_3_)_2_ to an equimolecular mixture of **Fc**′**Crypt**·NaCF_3_SO_3_ and **5**^+^PF_6_^−^ in acetonitrile led to the complex **5**^+^·2Zn^2+^, which is a strong oxidant and capable of oxidizing **Fc**′**Crypt**·Na^+^ quantitatively. The resulting **Fc**′**Crypt**^+^·Na^+^ subsequently displayed a drastically decreased affinity for Na^+^, resulting in quantitative removal of Na^+^ from **Fc**′**Crypt**^+^·Na^+^. In order to obtain the free molecule of **5**, a strong ligand cyclam was added to the reaction mixture, which was capable of removing Zn^2+^ ions irreversibly (Scheme 6). Free aminoferrocene ligand **5** further acted as a reducing agent to reduce **Fc**′**Crypt** ^+^ to **Fc**′**Crypt**, which finally regained its ability to bind Na^+^. The reactions shown in Scheme 6 could further be monitored by UV/Vis spectroscopy, where the absorption spectra of **5**, **5**^+^, **5**·2Zn^2+^, and **5**^+^·2Zn^2+^ displayed very distinctive signals [59].

In order to determine whether the reactions depicted in Scheme 6 actually took place and to further determine whether these reactions are kinetically feasible within a given time frame, ^1^H NMR and UV/Vis titration were performed. In both experiments, a solution of one equivalent of Zn(CF_3_SO_3_)_2_ in CH_3_CN was added stepwise to a mixture of **5**^+^PF_6_^−^ and **Fc**′**Crypt**·Na^+^ in CH_3_CN, followed by one equivalent of cyclam. Although the UV/Vis experiment is ideal for observing species associated with **5** and **5**^+^, due to the negligible extinction coefficients, it is not suitable for the detection of species derived from **Fc**′**Crypt**. To cover this gap, a ^1^H NMR titration experiment was performed, where CD_3_CN was used as solvent, and one equivalent of Zn^2+^ salt was found to be sufficient to initiate the reaction sequence, as shown in Scheme 6 [60].

#### 6.1.2. Ion Recognition Receptor

**Scheme 7:**
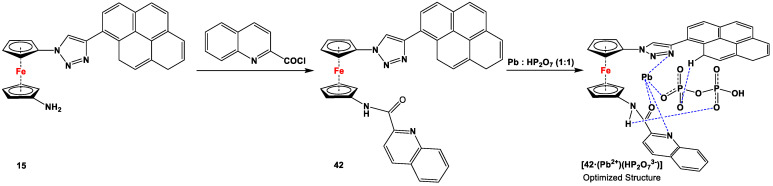
Synthesis of heteroditopic receptor **42** and its ion recognition (shown as blue dashed bonds) properties with Pb^2+^ and HP_2_O_7_^3−^ (1:1) [82].

Ferrocene–triazole and imidazole derivatives have found potential applications in the fields of electrochemical detection and sensing via host–guest chemistry [134]. N centers act as a Lewis bases and bind cations via inter- or intramolecular coordination, whereas anions are recognized through a complimentary C–H···anion or N-H···anion hydrogen bond formation for triazoles and imidazoles, respectively. Upon recognizing the cations, anions, or ion pairs, the resulting in situ-formed LM^+^, LA^−^, or LM^+^A^−^ complexes exhibit an easily detectable change in the redox potential of the ferrocene/ferrocenium redox couple, accompanied by perturbation of the emission signal in the emission spectrum [135]. Although such heteroditopic receptors for ion pair recognition involving organic triazoles are common [136,137], systems with ferrocene backbones are very rare [135,138,139]. Here it is noteworthy that Otón, Tárraga, and Molina et al. introduced an unsymmetrically substituted ferrocenylene triazole with sensing properties for unusual ion pair recognition [82]. In order to synthesize the sensor molecule, compound **15** was reacted with 2-quinaldoyl chloride to obtain species **42**, where one half of the ferrocene unit is linked to a pyrene through a 1,2,3-triazole and the other half is substituted by a quinoline ring, linked through amide linkage (Scheme 7).

Signal response of the emission for **42** in the presence of several anions (such as F^−^, Cl^−^, Br^−^, AcO^−^, NO_3_^−^, HSO_4_^−^, H_2_PO_4_^−^, and HP_2_O_7_^3−^ as TBA^+^ salts) was also studied, with only HP_2_O_7_^3−^ anion causing a small but significant change in the fluorescence spectrum. During the course of the titration, an isoemissive point at λ = 425 nm was conserved, which indicates the existence of an equilibrium between species **42** and complex [**42**·HP_2_O_7_^3−^]. The cross-selectivity of **42** was further tested with several cations (such as Li^+^, Na^+^, K^+^, Ca^2+^, Mg^2+^, Ni^2+^, Zn^2+^, Cd^2+^, Pb^2+^, Cu^2+^, and Hg^2+^), where only Pb^2+^ (ΔE_1/2_ = 75 mV) and Hg^2+^ (ΔE_1/2_ = 155 mV) displaying considerable perturbation in the oxidation wave, with a moderate amount of the cation (10 equivalents), while others required higher amounts (100 equivalents) or showed no changes at all. Upon testing the change in fluorescence spectra with the above-mentioned cations, it was revealed that only Hg^2+^ caused variations in the emission properties of receptor **42**, where a progressive decrease in the monomer emission intensity of about 85% (from Φ_F_ = 0.071 to Φ_F_ = 0.012) with the addition of 60 equivalents of Hg^2+^ was observed. When the ion pair recognition capability of **42** was studied via UV/Vis spectroscopy for Pb^2+^ and HP_2_O_7_^3−^ (1:1), a remarkable red shift in color was observed, where **42**, [**42**·Pb^2+^], [**42**·HP_2_O_7_^3−^], and [**42**·(Pb^2+^)(HP_2_O_7_^3−^)] displayed the visible colors of yellow, red, yellow, and green, respectively. In order to have a further insight into the structure of the resulting [**42**·(Pb^2+^)(HP_2_O_7_^3−^)] anion, theoretical calculations were used, and Pb···N^triazole^, Pb···O^HP2O7^, Pb···O^imide^, Pb-N^quinoline^, and O^HP2O7^···H^pyrene^ connectivities were observed in the optimized structure (see [**42**·(Pb^2+^)(HP_2_O_7_^3−^)] in Scheme 7).

The metal recognition properties of the imino-bridged [2.2]ferrocenophanes **18f** and **19d** were also evaluated by cyclic voltammetry, and a reversible electrochemical response was observed for Zn^2+^ complexation/decomplexation of **18f** [92]. Species **18f** further underwent altered oxidation in the presence of Cu^2+^ and Hg^2+^ cations, in contrast to Li^+^, Na^+^, K^+^, Mg^2+^, Ca^2+^, Cd^2+^, and Ni^2+^, for which no significant change in the corresponding electrochemical processes was found. Monitoring the recognition property of **18f** with UV/Vis spectroscopy, no observable changes were noticed upon addition of Li^+^, Na^+^, K^+^, Mg^2+^, Ca^2+^, Cd^2+^, and Ni^2+^, whereas significant changes in the absorption bands were observed upon addition of Cu^2+^, Hg^2+^, and Zn^2+^. With increasing amounts of Zn^2+^ added to **18f**, the low-energy (LE) metal-to-ligand transition band (MLCT) at λ = 491 nm gradually disappeared and a new band at λ = 600 nm progressively appeared, accompanied by a visible transformation in color from red to deep green. The presence of an isosbestic point at λ = 505 nm indicates a clean interconversion between the uncomplexed **18f** and complexed species **18f**·Zn^2+^. In sharp contrast to **18f**, species **19d** exhibited electrochemical responses only in the presence of Li^+^, accompanied by a red shift in the absorption signal from λ = 480 nm to λ = 669 nm and a clear isosbestic point located at λ = 614 nm. The exceptionally selective complexation behavior of **19d** can be explained by size selectivity, where, based on SCXRD of [**19d**·Li]B(C_6_H_5_)_4_, a small cavity is formed by two N atoms and Fe of bisiminophosphoranylferrocene, providing space up to the size of Li^+^ only.

**Scheme 8:**
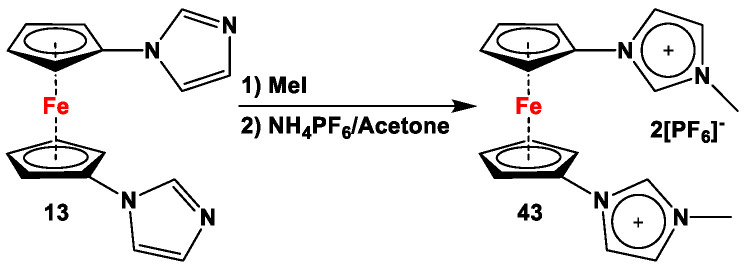
Synthesis of ferrocenylene bisimidazolium salts acting as Cl^−^ recognition receptor [80].

Jin and Liu et al. introduced a ferrocene-based receptor for the chloride anion **43**, (Scheme 8) which was prepared from compound **13** by stepwise reaction with MeI and NH_4_PF_6_. The addition of a sub-equivalent amount of Cl^−^ to receptor **43** caused a significant potential shift of ΔE_1/2_ = 310 mV, where the second oxidation wave overlapped with the oxidation wave of Cl^−^ in the CV curve. Upon addition of Cl^−^, the square wave voltammogram (SWV) curve of **43** showed a gradual anodic shift for the **43**·Cl^−^ complex, along with an increase in peak current. Upon addition of one equivalent of chloride, a clear two-wave potential was observed in SWV experiments, with a separation of ca. 160 mV from each other. Upon addition of more than two equivalents of Cl^−^, only the peak corresponding to the oxidation of the **43**·Cl^−^ complex was observed on the SWV curve, which implies a strong complexation between **43** and Cl^−^. When more than three equivalents of Cl^−^ were added to **43**, an additional peak for Cl^−^ oxidation (at ca. 0.7 V) was noticed [80].

#### 6.1.3. Mesoionic and Fischer-Type Carbenes

**Scheme 9:**
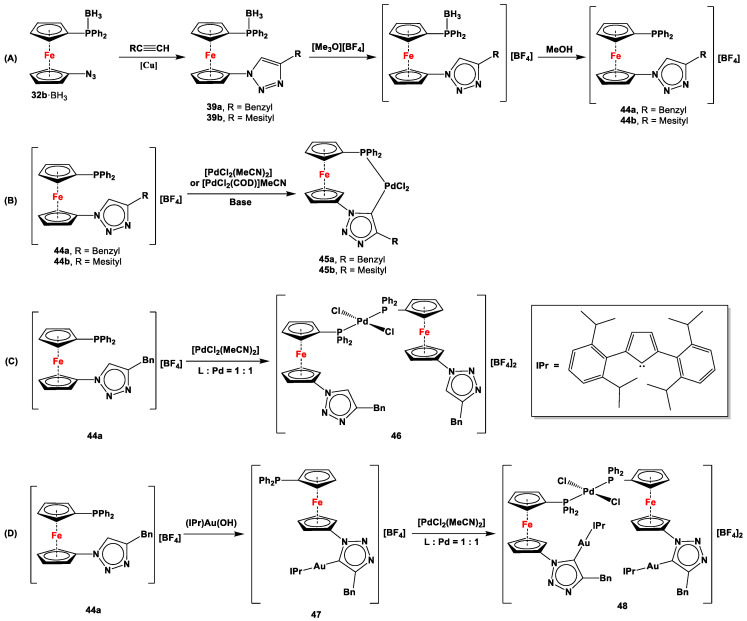
Mesoionic carbenes **39** (**A**) and their complexation with Pd(II) (**B**,**C**) and Au(II) (**D**), where IPr stands for 1,3-bis(2,6-diisopropylphenyl)imidazol-2-ylidene (inset) [122].

Mesoionic carbenes (MICs) are a type of a stable yet fairly reactive carbenoid intermediate that, despite being related to N-heterocyclic carbenes (NHCs), are an abnormal variant of the latter and therefore are sometimes referred to as remote N-heterocyclic carbenes [140]. MICs were notably introduced in the ferrocene system by Sarkar et al., where the resulting ferrocenyl heteromultimetallic iridium(I) and gold(I) complexes were used to demonstrate redox-switchable catalysis to synthesize oxazoline, furan, and phenols [141,142]. Unsymmetrically substituted ferrocenylene-based MICs were introduced by Štěpnička et al., where species **32b**·BH_3_ was first reacted with benzyl- and mesityl-substituted acetylene to obtain triazole **39** (Scheme 9A) [122]. After deprotonation with [Me_3_O][BF_4_] and subsequent removal of BH_3_, active carbenoids **44** was obtained, which was then reacted with transition-metal precursors to synthesize chelated and *η*^1^, *η*^1^-interbridged complexes **45** and **46**, respectively (Scheme 9B,C). When the Au-complex of **39a** (**47** with R = Bn) was further reacted with [PdCl_2_(MeCN)_2_], heterobimetallic gold and palladium complex **48** resulted (Scheme 9D) [122].

**Scheme 10:**
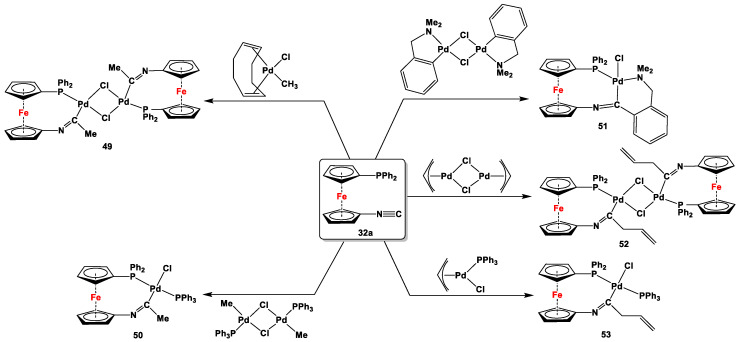
Fischer-type carbene complexes (**49**–**53**) with Pd(II), synthesized from unsymmetrically substituted compound **32b** [117].

Fischer-type carbenes are in their corresponding singlet states, often featuring an empty and accessible p_z_ orbital on the carbene C atom, which is capable of accepting π-back donation from the coordinating low-valent, late-transition elements [143]. The stability of such carbenes is further attained by the π-donor substituents in conjugation of the carbene atoms (e.g., alkoxy and alkylated amino groups). Although the concept of Fischer-type carbene complexes is well explored, with almost all transition metals and several organic moieties as backbones to host the molecule, Štěpnička and co-workers have recently reported synthesis and complexation for a unsymmetrically substituted ferrocenylene scaffold, where a second coordination from PPh_2_ unit helps to stabilize the metal cation (Pd^2+^) [117]. In order to synthesize these complexes, when **32a** was separately reacted with (cod)PdClMe, [(RR’)Pd(*μ*-Cl)]_2_ (where R = Me and R’ = PPh_3_, (RR’) = 2-(dimethylamino)methylphenyl, and (RR’) = *η*^3^-C_3_H_5_), (*η*^3^-C_3_H_5_)Pd(PPh_3_)Cl), simultaneous coordination of the Ph_2_P with Pd^2+^ and insertion of the isocyanide groups into the Pd–C bonds were observed (species **49**–**53**, Scheme 10).

#### 6.1.4. Dearomatization Reactions of N-Heterocycles

**Scheme 11:**
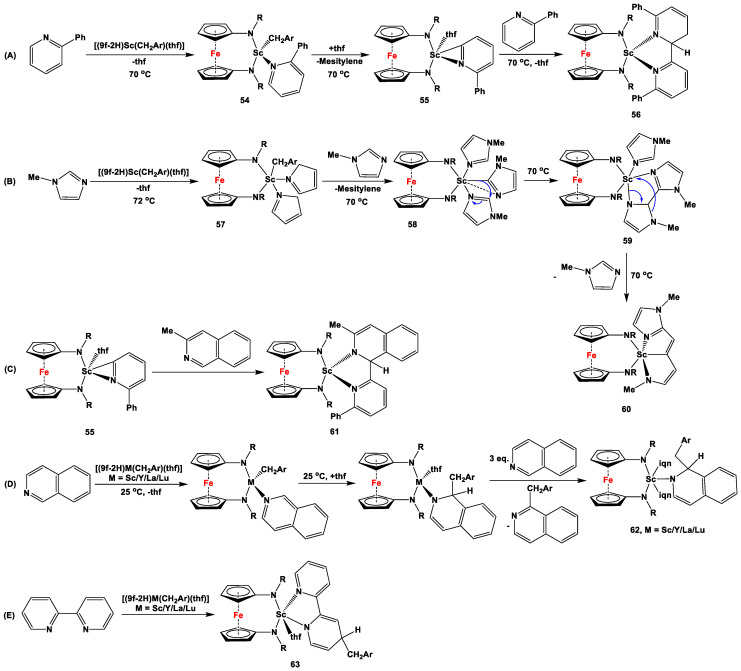
Proposed mechanistic details for dearomatization and ring-opening reactions (**A**–**E**) using [(**9f**-2H)M(CH_2_Ar)(THF)] (M = Sc, Y, La, Lu), where Ar, R, and iqn stand for 3,5-Me_2_C_6_H_3_, SiMe_2_*^t^*Bu, and isoquinoline, respectively [70,71,72,73,74].

Benzyl complexes of group 3 elements [(**9f**-2H)M(CH_2_Ar)(THF)] (where Ar = 3,5-Me_2_C_6_H_3_ and M = Sc, Y, La, and Lu), supported by a ferrocene diamide ligand **9f**, are reactive toward aromatic N-heterocycles via the coupling or breaking of C-N bonds (Scheme 11) [70,71,72,73,74]. For example, when a toluene solution of [(**9f**-2H)Sc(CH_2_Ar)(THF)] (where Ar = 3,5-Me_2_C_6_H_3_) was heated with 2-phenylpyridine at 70 °C, the first step of the reaction was commenced by the THF displacement and coordination of 2-phenylpyridine to form **54**, followed by ortho-metalation of the pyridine ring and simultaneous removal of mesitylene to produce the THF adduct **55** [70]. Upon prolonged heating at 70 °C in toluene solution, species **55** converted into C-C-coupled product **56**, where among the two pyridine rings, one was dearomatized (Scheme 11A). When species [(**9f**-2H)Sc(CH_2_Ar)(THF)] (where Ar = 3,5-Me_2_C_6_H_3_) was reacted with 1-methylimidazole, displacement of THF was followed by the formation of imidazole-coordinated intermediate **57**, which underwent simultaneous removal of mesitylene to produce C-H activation product **58** (Scheme 11B). In the next step, C-C coupling occurred between two neighboring imidazole units, followed by the dearomatization of one imidazole ring to yield intermediate **59**, which very rapidly arranged itself to produce final product **60** (Scheme 11B). When **55** was synthesized, isolated, and subsequently reacted with 8-methylisoquinoline, corresponding C-C-coupled product **61** with a dearomatized isoquinoline unit was obtained (Scheme 11C) [72]. On the other hand, upon reaction of isoquinoline or 2,2′-bipyridine with [(**9f**-2H)M(CH_2_Ar)(THF)] (where M = Sc, Y, La, or Lu, and Ar = 3,5-Me_2_C_6_H_3_), alkyl migration of the benzyl ligand onto the pyridine ring was facilitated, accompanied by the dearomatization of the corresponding N-heterocycle, to yield **62** and **63** (Scheme 11D,E) [73,74]. When a toluene solution of [(**9d**-2H)Lu(CH_2_Ar)(THF)_2_] was heated at 70 °C separately with 1-methylimidazole or isoquinoline, corresponding products similar to **60** and **62** were obtained (with R = adamantyl), respectively [74].

#### 6.1.5. Exploration of Oxidation Reactions of Germylenes

**Scheme 12:**
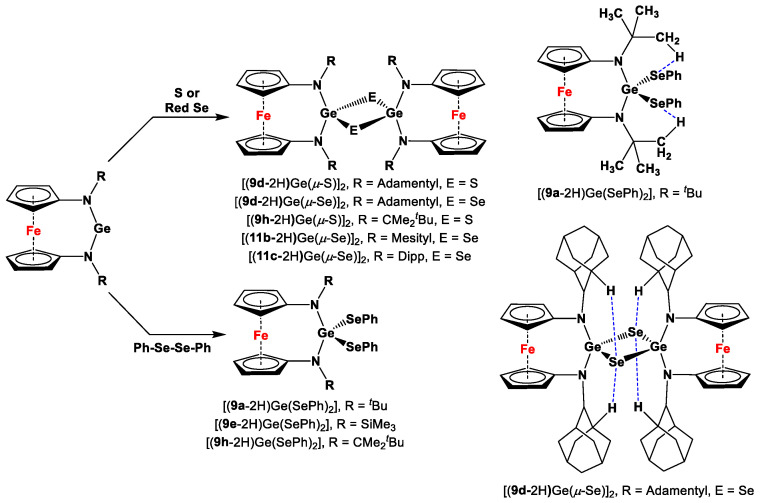
Oxidative addition reactions of germylenes and intramolecular CH···Se linkages (shown as blue dashed bonds) for [(**9a**-2H)Ge(SePh)_2_] and [(**9d**-2H)Ge(*μ*-Se)]_2_ [69].

Siemeling and co-workers recently explored the oxidization reaction on their flagship germylenes, prepared from 1,1′-diaza ferrocenes (**9a**, **9b**, **9d**, **9e**, **9h**, **11b**, and **11c**), where species (**9a**-2H)Ge, (**9d**-2H)Ge, (**9e**-2H)Ge, (**9h**-2H)Ge, (**11b**-2H)Ge, and (**11c**-2H)Ge were separately treated with elemental sulfur (S_8_), elemental selenium (red Se), and PhSe-SePh to obtain the oxidized products, such as [(**9a**-2H)Ge(SePh)_2_], [(**9d**-2H)Ge(*μ*-S)]_2_, [(**9d**-2H)Ge(*μ*-Se)]_2_, [(**9e**-2H)Ge(SePh)_2_], [(**9h**-2H)Ge(*μ*-S)]_2_, [(**9h**-2H)Ge(*μ*-Se)]_2_, [(**9h**-2H)Ge(SePh)_2_], [(**11b**-2H)Ge(*μ*-Se)]_2_, and [(**11c**-2H)Ge(*μ*-Se)]_2_ (Scheme 12). Unprecedentedly short intramolecular CH···Se distances were observed in SCXRD-analyzed structures of [(**9a**-2H)Ge(SePh)_2_] and [(**9d**-2H)Ge(*μ*-Se)]_2_ (Scheme 12) [69].

### 6.2. Catalytic Reactions

#### 6.2.1. Ring-Opening Polymerization (ROP) of Lactides and Cyclic Esters

**Scheme 13:**
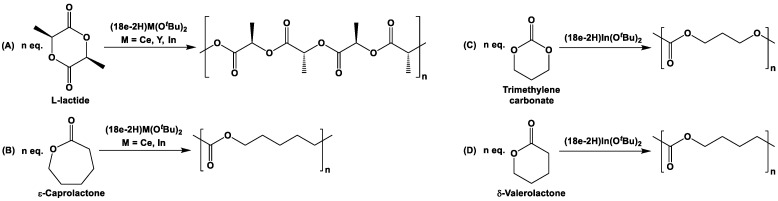
Polymerization of L-lactide (**A**), ε-caprolactone (**B**), trimethylene carbonate (**C**), and δ-valerolactone (**D**) [95,96].

In order to evaluate the catalytic activity for the ROP, (**18e**-2H)Ce(O*^t^*Bu)_2_ was reacted at 70 °C with L-lactide and ε-caprolactone (Scheme 13A,B) [95]. The reaction with 100 equiv. of ε-caprolactone took 4 h to reach 80% conversion, whereas a similar reaction with 100 equiv. of L-lactide required only 20 min. Although ROP of ε-caprolactone is generally more facile than that of L-lactide, the extraordinarily high reactivity of L-lactide is not fully understood. Here, it is to be noted that the isotactic polymer was formed exclusively without epimerization of the stereogenic centers (Scheme 13A). By comparison, the simple alkoxide Ce(O*^t^*Bu)_4_(THF)_2_ was found to be more active than (**18e**-2H)Ce(O*^t^*Bu)_2_ for L-lactide polymerization (Scheme 13A). When (**18e**-2H)Y(O*^t^*Bu)(THF) was used for L-lactide polymerization (Scheme 13A), it was observed that the Y-complex was more active than (**18e**-2H)Ce(O*^t^*Bu)_2_, with the ROP occurring at room temperature within minutes for the Y-counterpart. Molecular weight analyses of the polymers resulting from ROPs showed that the polymers from (**18e**-2H)Y(O*^t^*Bu)(THF) had lower poly-dispersity indices (PDIs) than those from (**18e**-2H)Ce(O*^t^*Bu)_2_. The Mulliken charges, calculated by DFT on (**18e**-2H)Y(O*^t^*Bu)(THF) and (**18e**-2H)Ce(O*^t^*Bu)_2_, indicated that the Y-center in (**18e**-2H)Y(O*^t^*Bu)(THF) is more electrophilic than the Ce-center in (**18e**-2H)Ce(O*^t^*Bu)_2_, making yttrium more reactive than cerium toward L-lactide [95]. However, unprecedentedly high reactivity towards the polymerization of L-lactide, ε-caprolactone, trimethylene carbonate, and δ-valerolactone was further achieved by ROP reaction with complex (**18e**-2H)In(O*^t^*Bu) at room temperature (Scheme 13) [96].

**Scheme 14:**
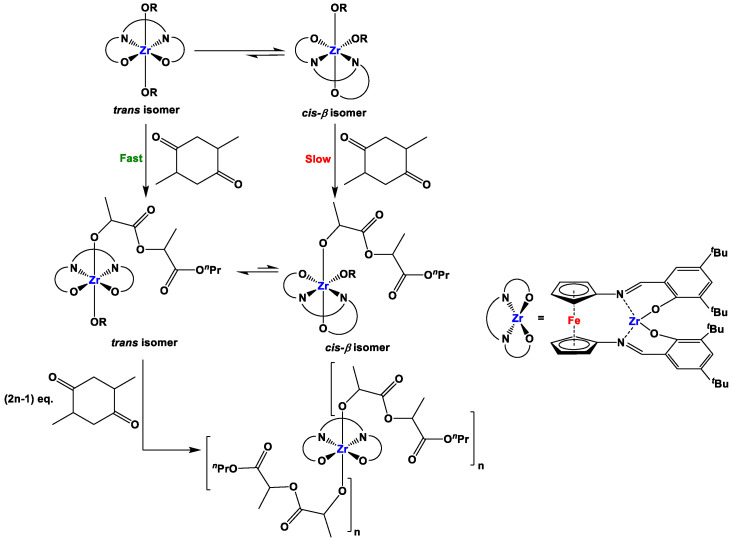
Proposed reactions of (**18e**-2H)Zr(OR)_2_ (where R = *^n^*Pr and *^i^*Pr) and lactide [98].

In order to explore the geometric change during lactide ring-opening polymerization, Diaconescu and co-workers used (**18e**-2H)Zr(O*^n^*Pr)_2_, (**18e**-2H)Zr(O*^i^*Pr)_2_, and (**18e**-2H)Zr(O*^t^*Bu)_2_ as precatalysts, where (**18e**-2H)Zr(O*^t^*Bu)_2_ showed no activity but both (**18e**-2H)Zr(O*^n^*Pr)_2_ and (**18e**-2H)Zr(O*^i^*Pr)_2_ enabled yields in the range of 60–70% at 100 °C over a reaction time of 24 h [98]. ^1^H NMR experiments of the reaction mixtures indicated that the corresponding reactions with (**18e**-2H)Zr(O*^n^*Pr)_2_ and (**18e**-2H)Zr(O*^i^*Pr)_2_ proceeded with a geometric change from *cis-β* to *trans* within 2 h upon heating at 100 °C. A similar geometric change was not observed with (**18e**-2H)Zr(O*^t^*Bu)_2_ even after 24 h heating at 100 °C, and consequently, catalysis did not occur for the latter. Here, it is noteworthy that (**18e**-2H)Zr(O*^n^*Pr)_2_, (**18e**-2H)Zr(O*^i^*Pr)_2_, and (**18e**-2H)Zr(O*^t^*Bu)_2_ contain *cis-β* and *trans* isomers in ratios of 71:29, 84:16, and 95:5 at room temperature [98]. ^1^H NMR experiments of the reaction mixtures further indicated that the polymerization majorly propagates after the previously mentioned change in geometry (i.e., *cis-β* to *trans*, Scheme 14). This observation further complies with the previously reported reactivity of salen-TiCl_2_ complexes [144,145,146].

#### 6.2.2. Redox-Switchable Catalysis

**Figure 6:**
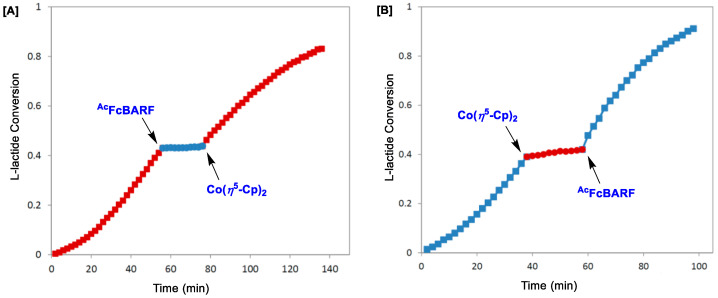
Plot of catalytic conversion vs. time (min) for the polymerization of (**A**) L-lactide with **8**·Zr(O*^t^*Bu)_2_, and (**B**) ε-caprolactone with [**8**·Zr(O*^t^*Bu)_2_]BARF, where ^Ac^FcBARF (^Ac^Fc = Fc(COCH_3_)) and Co(*η*^5^-Cp)_2_ were used as the oxidant and reductant, respectively. Adapted with permission from Ref [61]. Copyright 2014 American Chemical Society.

The activities of group 4 metals (Zr and Ti) for ROP of L-lactide and ε-caprolactone were further explored with redox-moderated precatalyst **8**·Zr(O*^t^*Bu)_2_, where oxidized and reduced forms of the corresponding metal complex affected the rate of polymerization [61]. When **8**·Zr(O*^t^*Bu)_2_ was heated at 100 °C in the presence of 100 equiv. of L-lactide, 90% conversion could be achieved within 2 h. On the other hand, with the oxidized version of **8**·Zr(O*^t^*Bu)_2_ as a catalyst (i.e., [**8**·Zr(O*^t^*Bu)_2_]BARF, synthesized via oxidation of **8**·Zr(O*^t^*Bu)_2_ with ^Ac^FcBARF, where ^Ac^Fc = Fc(COCH_3_)), <5% conversion was observed under the same reaction conditions as before. However, the activity toward ε-caprolactone showed the opposite trend, where [**8**·Zr(O*^t^*Bu)_2_]BARF and **8**·Zr(O*^t^*Bu)_2_ exhibited 98% and <5% conversion, respectively, with 100 equiv. of starting material at 25 °C over 24 h. In situ conversion between the oxidized and reduced forms of **8**·Zr(O*^t^*Bu)_2_ was further examined regarding their catalytic implications, where ^Ac^FcBARF was added to the reaction mixture at 43% conversion of L-lactide to polylactide (Figure 6A). Owing to the oxidation of **8**·Zr(O*^t^*Bu)_2_ to [**8**·Zr(O*^t^*Bu)_2_]BARF, the polymerization halted and resumed at the previous rate upon reduction with Co(*η*^5^-Cp)_2_ (Figure 6A). Similarly, when Co(*η*^5^-Cp)_2_ was added to the reaction mixture for the polymerization of ε-caprolactone with [**8**·Zr(O*^t^*Bu)_2_]BARF, the conversion halted until further oxidation (via the in situ addition of ^Ac^FcBARF) was performed (Figure 6B). Upon analyses with gel-permeation chromatography (GPC), the resulting polymers showed narrow molecular weight distribution with PDIs in the range of 1.1 to 1.2 [61].

By using these redox switches, Diaconescu and coworkers also demonstrated the successful syntheses of AB- and BA-type diblock and ABA- and ABC-type triblock copolymers [147]. For example, L-lactide was first polymerized in the presence of **8**·Zr(O*^t^*Bu)_2_, followed by in situ oxidation of **8**·Zr(O*^t^*Bu)_2_ with ^Ac^FcBARF and the addition of cyclohexene oxide to obtain diblock co-polymer [L-lactide]_a_-[cyclohexene oxide]_b_. Co(*η*^5^-Cp)_2_ was then added to the resulting reaction mixture, followed by the addition of *β*-butyrolactone to obtain ABC-type triblock copolymer [L-lactide]_a_-[cyclohexene oxide]_b_-[*β*-butyrolactone]_c_. When the mechanistic study for block-dependent copolymerization of cyclohexene oxide and lactide was performed for ring-opening polymerization, it was found that the reaction is thermodynamically unfavorable for lactide alone with [**8**·Zr(O*^t^*Bu)_2_]BARF [61,147,148]. However, this reaction becomes thermodynamically favorable for lactide after the polymerization of cyclohexene oxide with [**8**·Zr(O*^t^*Bu)_2_]BARF, where the initiation (or ring-opening) of lactide is thermodynamically favorable but the propagation is not. The propagation step for the polymerization of lactide is only possible after the polymerization of cyclohexene oxide [148].

**Figure 7:**
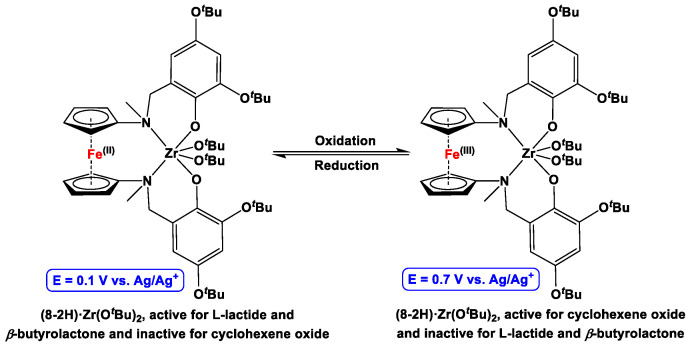
Electrochemically moderated one-pot polymerization with **8**·Zr(O*^t^*Bu)_2_ [149].

The same group further reported an electrochemically controlled synthesis of multiblock copolymers, where the redox state of the precatalyst (**8**-2H)·Zr(O*^t^*Bu)_2_ was electrochemically altered with a glassy carbon electrode, which resulted in a change in the catalytic selectivity of the catalyst [149]. For example, a sequential addition of L-lactide to a solution of TPANTf_2_ (75 mM; TPANTf_2_ = tetrapropylammonium bistriflimide) and 1,2-difluorobenzene (1.5 mL) of (**8**-2H)·Zr(O*^t^*Bu)_2_, followed by electrochemical oxidation and addition of cyclohexene oxide, yielded AB-type diblock copolymer [L-lactide]_a_-[cyclohexene oxide]_b_. When the resulting reaction mixture was further electrochemically reduced and L-lactide monomer was added, ABA-type triblock copolymer [L-lactide]_a_-[cyclohexene oxide]_b_-[L-lactide]_c_ resulted. The electrochemically controlled redox reaction of (**8**-2H)·Zr(O*^t^*Bu)_2_ ⇌ (**8**-2H)^+^·Zr(O*^t^*Bu)_2_, along with their corresponding bulk electrolysis potentials (vs. Ag/Ag^+^ pseudoreference electrode), are demonstrated in Figure 7.

**Figure 8:**
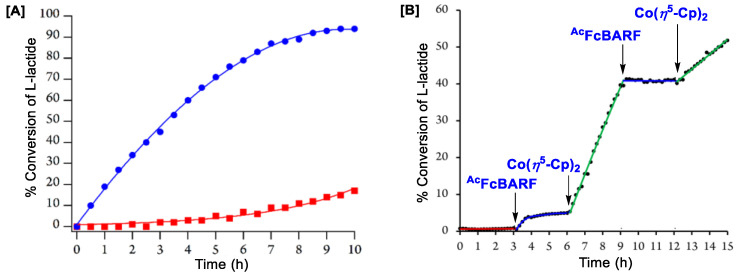
Plot for polymerization (%) of L-lactide with reaction time (h), (**A**) where red and blue markers have been used for catalysis with (**18e**-2H)Ti(O*^i^*Pr)_2_ and [(**18e**-2H)Ti(O*^i^*Pr)_2_]BARF, respectively, (**B**) and in situ redox-switching with (**18e**-2H)Ti(O*^i^*Pr)_2_ as starting precatalyst. Adapted with permission from Ref. [93]. Copyright 2015 American Chemical Society.

Polymerization of L-lactide was further performed at 90 °C with (**18e**-2H)Ti(O*^i^*Pr)_2_ and its oxidized version, [(**18e**-2H)Ti(O*^i^*Pr)_2_]BARF (synthesized via oxidation of (**18e**-2H)Ti(O*^i^*Pr)_2_ with ^Ac^FcBARF), and the catalytic conversion was plotted against time, with (**18e**-2H)Ti(O*^i^*Pr)_2_ showing an extraordinarily low catalytic conversion rate (red markers in Figure 8A) [93]. This observation is opposite to the previously reported catalytic trend, where electron deficient complexes showed substantially lower conversion rates than their corresponding electron-rich counterparts [61,150]. To examine the redox-switching ability, (**18e**-2H)Ti(O*^i^*Pr)_2_ was reacted with 100 equiv. of L-lactide, where the oxidation state of the catalyst was in situ modulated via the addition of ^Ac^FcBARF and Co(*η*^5^-Cp)_2_ as the oxidant and reductant, respectively. As shown in Figure 8B, the catalytic activity of (**18e**-2H)Ti(O*^i^*Pr)_2_ was substantially low until the complex is in situ oxidized with ^Ac^FcBARF, but subsided after reaching ca. 4–6%. When the oxidized catalyst was further in situ reduced upon addition of Co(*η*^5^-Cp)_2_, the catalyst surprisingly began to perform at a greater rate until up to ca. 40% conversion [93]. Further in situ oxidation with ^Ac^FcBARF halted the catalytic activity, which was followed by restoration of the same upon addition of Co(*η*^5^-Cp)_2_. As the trend in the in situ redox-switchable catalysis (Figure 8B) is different than that found in Figure 8A, the in situ oxidation of (**18e**-2H)Ti(O*^i^*Pr)_2_ was performed with ^Ac^FcBARF in the presence of excess L-lactide, which showed a halted reactivity at ca. 4–6% catalytic conversion. Upon subsequent in situ reduction of the resulting oxidized species with Co(*η*^5^-Cp)_2_ in the presence of excess L-lactide, a dramatic increase in polymeric activity could be observed, with the conversation reaching up to ca. 80% over 5 h. As an outcome of the previous observations, Long and coworkers concluded that the catalytic activities of (**18e**-2H)Ti(O*^i^*Pr)_2_ and [(**18e**-2H)Ti(O*^i^*Pr)_2_]BARF not only depend on the oxidation states of the metal ions in the respective precatalysts, but also considerably depend on the chemical species present during their catalytic reactions.

**Scheme 15:**
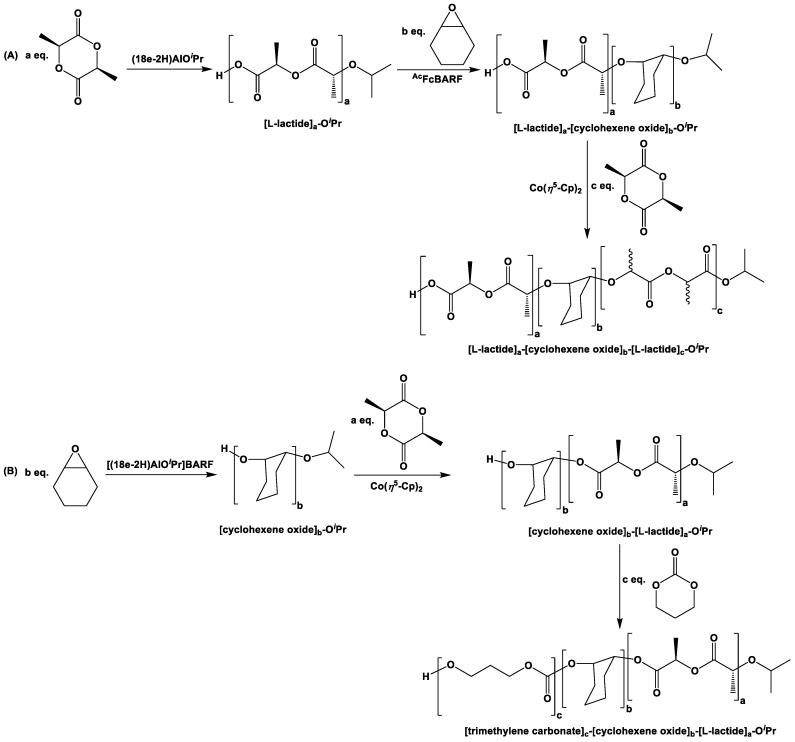
ABA- (**A**) and ABC-type (**B**) triblock polymers, synthesized with redox-switchable catalyst (**18e**-2H)AlO*^i^*Pr [94].

*Iso*-propoxide complexes of aluminum, supported by **18e** (i.e., (**18e**-2H)AlO*^i^*Pr and [(**18e**-2H)AlO*^i^*Pr][BARF]), were further used to examine the redox switchability for the ring-opening polymerization of L-lactide, ɛ-caprolactone, δ-valerolactone, β-butyrolactone, trimethylene carbonate, and cyclohexene oxide, where only the non-oxidized compound (i.e., (**18e**-2H)AlO*^i^*Pr) was found to be active for L-lactide, β-butyrolactone, and trimethylene carbonate [94]. Although 64% and 98% conversion were observed after 24 h at 100 °C (catalyst:monomer, 1:100), for L-lactide and β-butyrolactone, respectively, a quantitative conversion was observed for trimethylene carbonate (catalyst:monomer, 1:100) even after 2.5 h at room temperature. In the case of ɛ-caprolactone and δ-valerolactone (catalyst:monomer, 1:100), no difference in the activity of oxidized and reduced forms of the catalyst could be observed, as in both cases the quantitative transformation could be achieved within 2 h at room temperature. On the other hand, when similar ring-opening polymerization was investigated for cyclohexene oxide separately, with reduced and oxidized forms of the above-mentioned catalyst (catalyst:monomer, 1:100), only the oxidized form of the catalyst (i.e., [(**18e**-2H)AlO*^i^*Pr][BARF]) was found to be active [94]. By using the selectivity for the catalytic reactions of the above-mentioned monomers, the syntheses of AB block copolymers were attempted with L-lactide and cyclohexene oxide. In order to do so, polymerization of L-lactide was first performed with catalyst (**18e**-2H)AlO*^i^*Pr, followed by the addition of ^Ac^FcBARF and cyclohexene oxide to stop the L-lactide polymerization and initiate the corresponding polymerization of cyclohexene oxide to obtain [L-lactide]_a_-[cyclohexene oxide]_b_-O*^i^*Pr (Scheme 15A). The reverse diblock co-polymer [cyclohexene oxide]_b_-[L-lactide]_a_-O*^i^*Pr could further be synthesized via the following steps: initial polymerization of cyclohexene oxide with [(**18e**-2H)AlO*^i^*Pr][BARF], followed by the addition of Co(*η*^5^-Cp)_2_, along with L-lactide (Scheme 15B). Triblock ABA co-polymer [L-lactide]_a_-[cyclohexene oxide]_b_-[L-lactide]_c_-O*^i^*Pr was synthesized from [L-lactide]_a_-[cyclohexene oxide]_b_-O*^i^*Pr via subsequent in situ reduction of [(**18e**-2H)AlO*^i^*Pr][BARF] with Co(*η*^5^-Cp)_2_, followed by the addition of L-lactide monomers to the reaction mixture (Scheme 15A) [94]. Similarly, when trimethylene carbonate was added at 100 °C after the formation of [cyclohexene oxide]_b_-[L-lactide]_a_-O*^i^*Pr, the triblock co-polymer [trimethylene carbonate]_c_-[cyclohexene oxide]_b_-[L-lactide]_a_-O*^i^*Pr was obtained (Scheme 15B) [94].

**Scheme 16:**
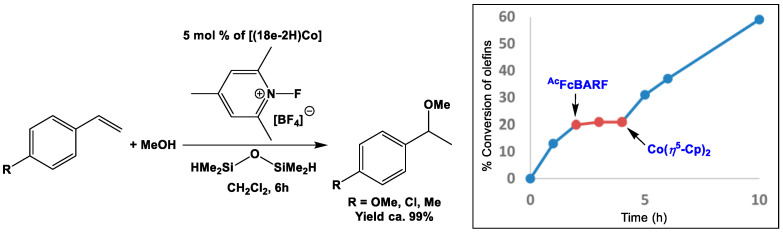
Catalytic reaction by [(**18e**-2H)Co] and plot of conversion versus time of the redox switch (inset). Adapted with permission from Ref. [97]. Copyright 2016 American Chemical Society.

Markovnikov hydroalkoxylation of unactivated olefins with cobalt complexes of salen-ligands accompanied by silane and N-fluoropyridinium salt was primarily reported by Hiroya and co-workers [151]. Inspired by the work of Hiroya et al. [151], Diaconescu and coworkers optimized the catalytic activity of [(**18e**-2H)Co] towards hydroalkoxylation of olefins in presence of siloxane TMDSO (TMDSO = HMe_2_Si-O-SiMe_2_H) and electrophilic fluorinating agent NFPBF_4_ (where NFPBF_4_ = N-fluoro-2,4,6-trimethylpyridiniumtetrafluoroborate) in CH_2_Cl_2_ (Scheme 16) [97]. Although this catalytic system was effective (yield ca. 99%) for many different varieties of styrene derivatives (Scheme 16), little to no activity was observed for alkyl or norbornyl derivatives. Moreover, when the tetrameric Co- and monomeric Zn-complexes were used in place of [(**18e**-2H)Co], little and no catalytic conversion were observed for [(**18e**-2H)Co]_4_ and [(**18e**-2H)Zn], respectively. In situ oxidation of [(**18e**-2H)Co] by addition of ^Ac^FcBARF halted the catalytic reaction, which further resumed to the previous rate upon in situ reduction with Co(*η*^5^-Cp)_2_ (Scheme 16).

#### 6.2.3. Pd(II)-Catalyzed Cross-Coupling Reactions

**Scheme 17:**
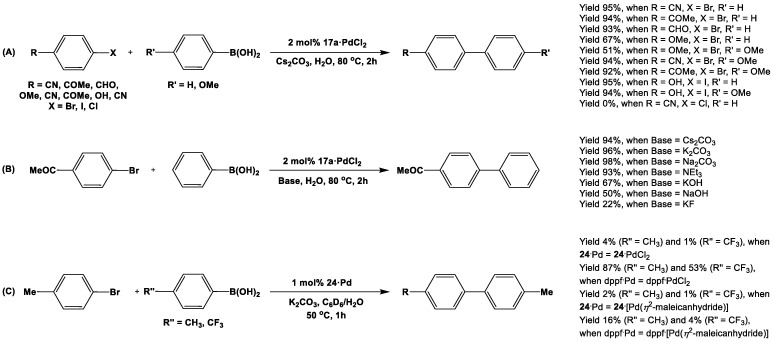
Suzuki cross-coupling reactions with catalysts **17a**·PdCl_2_ (**A**), **24**·PdCl_2_ (**B**), and **24**·[Pd(*η*^2^-maleic anhydride)] (**C**) [88,110].

Phosphine ligands have been employed for Pd(II)-catalyzed Suzuki cross-coupling of haloarenes with arylboronic acid for decades [152]. Being interested in developing a new generation of non-poisonous, environment-friendly, water-based, and phosphine-free catalysts of high efficiency, Hor and coworkers investigated the catalytic efficiency of the Pd(II)-complex of 1,1′-diiminoferrocene **17a** for such cross-coupling reactions [88]. As the products were water-insoluble, their easy separation and isolation from the crude reaction mixture provided an additional advantage for this catalyst (Scheme 17A). Although **17a**·PdCl_2_ has successfully catalyzed cross-coupling reactions between aryl bromides/iodides and aryl boronic acids in non-homogenous aqueous reaction conditions, it failed to display any catalytic activity for Cl-substituted starting materials (Scheme 17A). The choices of base (Scheme 17B), catalytic load, and recoverability have further been investigated for **17a**·PdCl_2_ by Hor et al [88]. 

In order to compare the catalytic activity of 1,1′-distibanylferrocene **24** with dppf, Štěpnička et al. used **24**·PdCl_2_ and **24**·[Pd(*η*^2^-maleic anhydride)] as catalysts for Suzuki cross-coupling reactions [110]. However, the yields for reactions with Pd(II)-complexes of **24** (i.e., **24**·PdCl_2_ and **24**·[Pd(*η*^2^-maleicanhydride)]) were rather small compared to those from respective complexes of dppf (i.e., dppf·PdCl_2_ and dppf·[Pd(*η*^2^-maleic anhydride)], Scheme 17C).

**Scheme 18:**
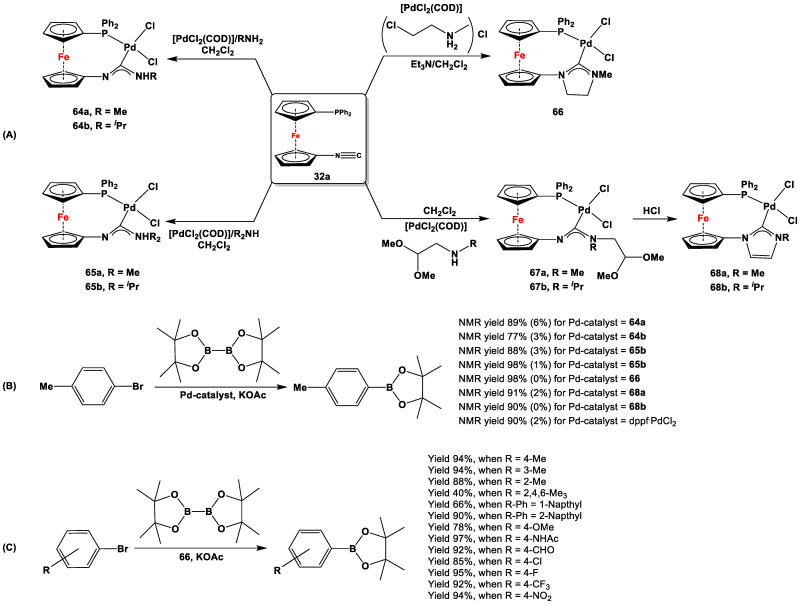
Syntheses (**A**) and Miyaura borylation reactions (**B**,**C**) with precatalysts **64**–**66** and **68** [118]. The yields of 4,4′-dimethylbiphenyl are given in parentheses (**B**).

In order to explore the Miyaura borylation reaction with Pd(II)-complexes of 1,1′-aminophosphanylferrocene carbene ligands, **64**–**68** were first synthesized from **32a** via reaction with PdCl_2_(COD) and primary or secondary amines or ammonium salt (Scheme 18A) [118]. Precatalysts **64**–**66** and **68** were then reacted with 4-bromotoluene and bis(pinacolato)diborane to synthesize the corresponding boronic esters (Scheme 18B) [118]. A series of optimization experiments with different solvents and bases revealed *^i^*PrOH and KOAc to be suitable for these reactions. When **64**–**66** and **68** were further used as precatalysts for the Miyaura borylation reaction of 4-bromotoluene, **65b** and **66** showed the maximum catalytic activity and selectivity, resulting in ca. 98% yield of boronic ester and 0% yield of homocoupled product 4,4′-dimethylbiphenyl (based on NMR spectra measured from the reaction mixture, Scheme 18B) [118]. When the most synthetically accessible complex, **66**, was used as catalyst for reactions with several other aryl bromides, the lowest coupling yields could be observed for mesityl bromide (Scheme 18C). Nonetheless, the reactions with other bromides resulted in decent to excellent yields, varying in a range of 66–97% (Scheme 18C).

#### 6.2.4. Au(I)-Catalyzed Annellation Reactions

**Scheme 19:**
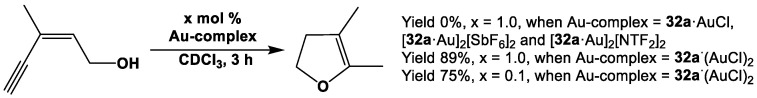
Au-catalyzed cycloisomerization of enynol [113].

Being inspired by the catalytic properties of gold(I) complexes of Fc′(PPh_2_)(CN) [153], complexes Fc′(Ph_2_P·AuCl)NC (i.e., **32a**·AuCl); Fc’(Ph_2_P·AuCl)(NC·AuCl) (i.e., **32a**·(AuCl)_2_)); and *η*^1^, *η*^1^-interbridged complexes [Fc′(Ph_2_P·Au)NC]_2_[SbF_6_]_2_ (i.e., [**32a**·*μ*-Au]_2_[SbF_6_]_2_) and [Fc′(Ph_2_P·Au)NC]_2_[NTF_2_]_2_ (i.e., [**32a**·*μ*-Au]_2_[NTF_2_]_2_) were used as catalysts for cycloisomerization reaction of enynol by Štěpnička et al. [113]. Owing to very strong Au-CN bonds in the dimeric complexes, formation of the catalytically active mono-gold species was suppressed, and consequently, no substantial yields of 2,3-dimethylfuran were observed for [**32a**·*μ*-Au]_2_[SbF_6_]_2_ or [**32a**·*μ*-Au]_2_[NTF_2_]_2_ (Scheme 19). Although mono-gold species **32a**·AuCl was found to be ineffective for catalysis, di-gold species **32a**·(AuCl)_2_ demonstrated the highest catalytic yield of 75–89% after 3 h (Scheme 19).

**Scheme 20:**
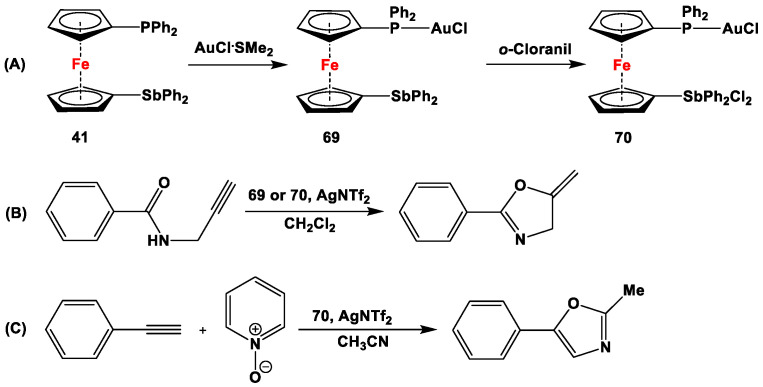
Syntheses (**A**) and AgNTf_2_-activated cyclization of *N*-propargylbenzamide (**B**,**C**) by complexes **69** and **70** [126].

Au(I)-complexes derived from ligand **41** (i.e., **69** and **70**, following reactions depicted in Scheme 20A) have further been used for in situ AgNTf_2_-activated cyclization of *N*-propargylbenzamide to produce 4,5-dihydro-5-methylene-2-phenyloxazole (Scheme 20B) [126]. Although the yield obtained with phosphine complex **69** was very high (ca. 97% NMR yield), the initial acceleration of the reaction, followed by catalyst decomposition, was observed for the analogous reaction with complex **70**. Moreover, complexes **69** and **70** have further been used for Au-catalyzed oxidative [2 + 2 + 1] cyclization of ethynylbenzene with acetonitrile (Scheme 20C), with **69** producing a higher yield (37%) than **70** (27%). As the previous records demonstrated, the outcome of such catalytic reactions is dependent on the *N*-oxide [154]. Štěpnička and co-workers have further reported the catalytic yields using several different *N*-oxides with complex **69**, where the substituted (with 4-Me, 4-OMe, and 4-NO_2_) pyridine *N*-oxides produced substantially lower yields (3–23%) than the unsubstituted one (37%). When sterically demanding *N*-oxides (such as 2,4-Me_2_-pyridine *N*-oxide, 1,5-Me_2_-pyridine *N*-oxide, 8-methylquinoline *N*-oxide, and 2-methylquinoline *N*-oxide) were compared in this reaction, the highest yield was obtained with 8-methylquinoline *N*-oxide (73%), whereas the other sterically encumbered species produced yields in the range of 11–28% for the reaction demonstrated in Scheme 20C [126].

**Scheme 21:**
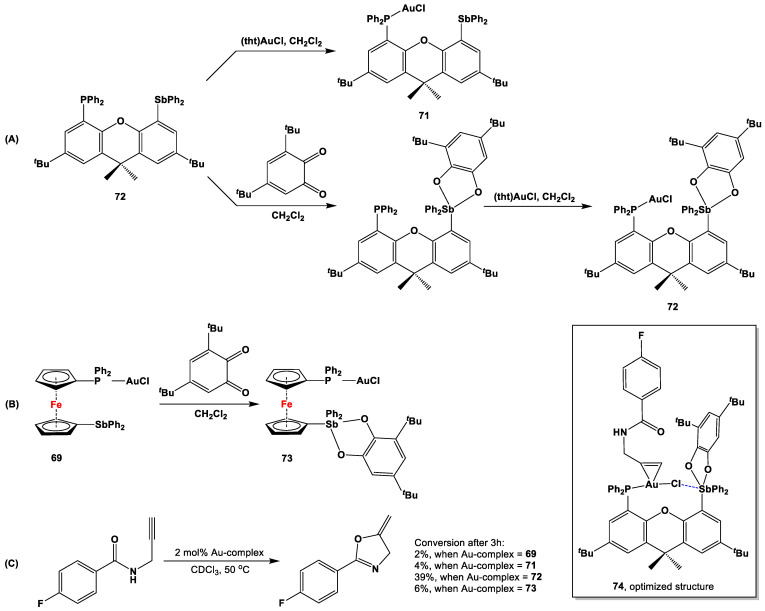
Syntheses (**A**,**B**) and catalytic reactions with precatalysts **69**, **71**–**73** (**C**), and the computationally optimized structure of adduct **74** for cycloisomerization of 4-fluro-*N*-propargylbenzamide with precatalyst **72** [127].

Owing to their inherent carbophilic nature, Au(I) complexes have frequently been used as catalysts for various functionalization reactions with C=C and C≡C bonds [155]. In order to test their carbophilic nature for annellation reactions of 4-fluoro-*N*-propargylbenzamide, four Au(I) complexes of phosphanylstibanyl ligands (i.e., **69** and **71**–**73**) were synthesized as outlined in Scheme 21A,B [127]. While complexes **69**, **71**, and **73** were almost ineffective for catalysis, an onset of product formation could be observed right after the addition of **72** (Scheme 21C). This observation directly supports the effectiveness and increased carbophilicity of the AuCl center in **72**, where the Sb(V) center potentially engaged with the Au-Cl bond. On the other hand, as the ferrocenylene system freely rotated around the *η*^5^-Cp^center^—Fe—*η*^5^-Cp^center^ axis, species **73** attained a pre-organized orientation prior catalysis, which minimized the scope for the formation of AuCl→Sb(V) linkage. As a result, despite containing an Sb(V) center, species **73** showed only a modicum of catalytic activity (Scheme 21C). In order to explain the above catalytic results, Gabbaï and coworkers computed the structure of the presumed adduct **74**, formed between precatalyst **72** and the alkenyl substrate. The computationally optimized structure of adduct **74** displayed coordination of the alkyne to the Au center, with a simultaneous formation of an AuCl→Sb(V) linkage (shown by the blue dashed bond in the inset of Scheme 21), with the Au center being more exposed and consequently more active towards electrophilic addition.

## 7. Perspectives

In contrast to readily and commercially available dppf and its bulky bisphosphanyl ferrocene analogs (e.g., 1,1′-bis(di-tert-butylphosphanyl)ferrocene), their non-phosphanyl counterparts are relatively unexplored. Nevertheless, the so-far reported investigations indicate their application potential for redox-responsive molecular switches, ligands, and ion-recognition receptors. Although a new variety of redox-switchable catalysts for ring-opening polymerization of cyclic lactides and lactones could be developed from diamino-substituted ferrocenylenes, the catalytic activities for their distibanyl and mixed phosphanylstibanyl counterparts fall short compared to dppf for cross-coupling reactions. One of the key features for diamino-substituted ferrocenylenes is their ability to host (hetero)carbenes, which allow for the exploration of ferrocene-bridged N-heterocyclic systems with low-coordinate group 14 elements in the *ansa*-bridge. Intermetallic interactions (Fe→M) have further been explored within the framework of the ferrocenylene scaffolds, featuring extreme Fe→M distances with mixed N and P donor sites, based on experimental and computational evidence. The readily accessible mesoionic and Fischer-type carbenes further highlight the relevance of the mixed P,N-substituted ferrocenylene scaffold. Overall, pnictogen-substituted non-phosphanyl ferrocenes have found their major chemical impact in non-catalytic sectors, but their catalytic potential is just starting to emerge, and consequently, provides the scope for future investigation and development.

## Supplementary Materials

The following supporting information (SI) can be downloaded at: https://www.mdpi.com/article/10.3390/molecules29225283/s1, Table S1: Survey of SCXRD characterized complexes and compounds from 1,1’-bispnictogen substituted dppf-analogs, Table S2: Molecular parameters of cationic Ni(II), Pd(II), Sc(III), Lu(III) complexes of dppf and its 1,1’-diamino-, selected 1,1’-bisphosphanyl- and 1,1’-aminophosphanylferrocenes with Fe→Pd bonding interactions, Table S3: Survey of oxidation potentials (EO) for 1,1’-bispnictogen substituted dppf analogs and their complexes with potentials converted to the (C_5_Me_5_)_2_Fe/(C_5_Me_5_)_2_Fe^+^ scale. Other than the so-far cited references, a few additional reports have further been cited in the SI file [156,157,158,159,160,161,162,163,164,165,166].
