# Supplementary material for: 1,1′-Disubstituted Ferrocene Ligand Scaffolds Featuring Pnictogens Other than Phosphorus as Donor Sites
Source: Molecules. 2024 Nov 8;29(22):5283. doi: 10.3390/molecules29225283 (PMC11596527; doi:10.3390/molecules29225283)
Supplement: Supplementary file 1 [file molecules-29-05283-s001.zip › molecules-3242037-supplementary.pdf]

Table of Contents

Table S1. Survey of SCXRD characterized complexes and compounds from 1,1'-bispnictogen substituted dppf-analogs. 2

Open-bridged complexes (**A**, Figure 2) 2

Quasi-closed bridged complex (**B**, Figure 2) 2

Double bridged complexes (**C**, Figure 2) 2

η[1], η[1]-interbridged complexes (**D**, Figure 2) 2

Higher order η[1], η[1]-interbridged complex (**E**, Figure 2) 2

Chelated complexes for comparison (**F**, where E = P, Figure 2) 2

Chelated complexes (**F**, Figure 2) 2

Double chelated complex (**G**, Figure 2) 3

Annellated species with transition metal bridges (**H**, Figure 2) 3

Multidentate chelated species with main-group elements, transition metals and lanthanide bridges [**I** (Entries 58-78) and **J** (Entries 79-81), Figure 2] 3

Higher order species with intermolecular N-M-N bridges (M = Co, Zn) (**K**, Figure 2) 4

Carbenes, silylenes, germylenes, and stannylenes of 1,1′-diaminoferrocenes (**L**, Figure 2) 4

Compounds obtained by oxidation from **24** (**M** and **N**, Figure 2) 5

Table S2. Molecular parameters of cationic Ni(II), Pd(II), Sc(III), Lu(III) complexes of dppf and its 1,1'-diamino-, selected 1,1'-bisphosphanyl- and 1,1'-aminophosphanylferrocenes with Fe→Pd bonding interactions. 5

Cationic Pd(II) complexes with dppf-analogs for comparison 5

Cationic Pd(II) complexes for 1,1′-diaza- and 1,1′-azaphosphaferrocenes 5

Cationic Ni(II) complexes for comparison dppf-analogs for comparison 6

Cationic Ni(II) complexes 6

Complexes with Lu (III), Sc (III), Y (III), La (III) 6

Table S3. Survey of oxidation potentials (EO) for 6

Oxidation potentials (E°) for comparison 6

Oxidation potentials (E°) for 1,1′-bispnictogen substituted dppf analogs 7

Oxidation potentials (E°) for coordination complexes 7

Oxidation potentials (E°) for compounds with metal bridges, carbenes and germylenes 7

Compounds obtained by oxidation of **24** 8

Table S1. Survey of SCXRD characterized complexes and compounds from 1,1'-bispnictogen substituted dppf-analogs.

	Complexes	Dihedral angles (α)*, #	Bite angles (β_n)#	Distances between donor and acceptor atoms (E-M)#	Ref.
Open-bridged complexes (**A**, Figure 2)					
1	**5**·$(ZnBr_2)_2$	NA	NA	2.493(5) Å (N-Zn)	[1]
2	**24**·[Ru(η^6-1-Me,3-^{i}Pr-C_6H_4)Cl_2]$_2$	NA	NA	2.5969(4) Å (Sb-Ru)	[2]
3	**24**·[Rh(η^5-C_5Me_5)Cl_2]$_2$	NA	NA	2.5677(4) Å (Sb-Rh)	[2]
4	**24**·$(AuCl)_2$	NA	NA	2.4910(7) Å (Sb-Au)	[2]
5	**32a**·AgCl	NA	NA	2.4529(7) Å (P-Ag)	[3]
6	**32a**·AuCl	NA	NA	2.533(6)° (P-Au)	[3]
7	**32a**·$[AuCl]_2$	NA	NA	2.230(1)° (P-Au) and 1.930(5)° (C-Au)	[3]
8	**33**·[Ru(η^6-1-Me,3-^{i}Pr-C_6H_4)Cl_2]	NA	NA	2.3505(5) Å (P-Pd)	[4]
9	**33**·[Rh(η^5-C_5Me_5)Cl_2]	NA	NA	2.3218(8) Å (P-Pd)	[4]
10	(**34a** + H)·$PdCl_3$	NA	NA	2.2369(6) Å (P-Pd)	[5]
Quasi-closed bridged complex (**B**, Figure 2)					
11	(**24**)$_2$·(μ-$AgOClO_3$)$_2$	4.74°	NA	2.6520(3) Å (Sb-Ag)	[2]
Double bridged complexes (**C**, Figure 2)					
12	[**32a**·Ag($OCMe_2$)]$_2$[SbF_6]$_2$	2.4°	NA	2.385(1) Å (P-Ag)	[3]
13	[**32a**·Au($OCMe_2$)]$_2$[SbF_6]$_2$	2.6°	NA	2.5017(7) Å (P-Au)	[3]
η^1, η^1-interbridged complexes (**D**, Figure 2)					
14	(**33**)$_2$·$PdCl_2$	NA	NA	2.3488(6) Å (P-Pd)	[4]
15	[(**34a**)$_2$·$PdCl_2$]$(SbF_6)_2$	NA	NA	2.325 Å (P-Pd)	[5]
16	[(**34a**)$_2$·(μ-Pd_2Cl_4)]$(SbF_6)_2$	NA	NA	2.232(2) Å (P-Pd)	[5]
Higher order η^1, η^1-interbridged complex (**E**, Figure 2)					
17	[(**32a**)$_2$·Ag]$_2$$(SbF_6)_2$	4.0°	NA	2.5376(9) Å (P-Ag)	[3]
Chelated complexes for comparison (**F**, where E = P, Figure 2)					
18	dppf·Pd(η^2-C_{60})	2.3°	103.30(9)°	2.286(3) Å (P-Pd)	[6]
19	dppf·$PdCl_2$	6.8°	97.98(4)° and 97.8(2)°	2.262(4) Å (P-Pd)	[7]
20	dppf·Pt(η^2-C_{60})	3.3°	102.96(5)°	2.2700(13) Å (P-Pt)	[6]
21	dppf·$PtCl_2$	5.0°	91.6(2)°	2.266(5) Å (P-Pt)	[7]
22	Fc'($PMes_2$)(P^tBu_2)·$PdCl_2$	2.7°	103.37(5)°	2.3167(13) Å (P^{Mes}-Pd) and 2.352(2) Å (P^{tBu}-Pd)	[8]
Chelated complexes (**F**, Figure 2)					
23	**17a**·$PdCl_2$	7.3°	84.5(4)°	2.056 Å	[9]
24	**17a**·PdClMe	4.5°	90.2(4)°	2.030(13) Å and 2.200(10) Å (N-Pd)	[10]
25	**17i**·$MnCl_2$	8.4°	109.82(6)°	2.1493(12) Å (N-Mn)	[11]
26	**17i**·$FeCl_2$	11.3°	103.56(4)°	2.0626(11) Å (N-Fe)	[11]
27	**17i**·$FeCl_2(SbF_6)$	2.4°	114.60(9)°	1.925(2) Å (N-Fe)	[11]
28	**17i**·$CoCl_2$	11.6°	105.16(5)°	2.0310(14) Å (N-Co)	[11]
29	**17i**·$NiCl_2$	12.7°	103.47(6)°	2.015(1) Å (N-Ni)	[12]
30	**17i**·$CuCl_2$	11.3°	103.38(10)°	2.071(3) Å (N-Cu)	[11]
31	**17i**·$ZnCl_2$	11.6°	106.54(6)°	2.0280(15) Å (N-Zn)	[11]
32	**17i**·$PdCl_2$	11.8°	96.4°	2.088 Å (N-Pd)	[12]
33	(**20**)$_2$·(μ-$NiBr_2$)	1.0°	84.36(4)°	2.563(2) Å (As-Ni)	[13]
34	**22**·Pt(η^2-C_{60})	1.4°	100.12(3)°	2.3874(9) Å (As-Pt)	[6]
35	**22**·Pt(η^2-C_{70})	1.6°	100.61(5)°	2.3786(15) Å (As-Pt)	[6]

36	**24**·[Ru(η^5-C_5Me_5)Cl]	5.8°	92.48(2)°	2.5578(8) Å (Sb-Ru)	[2]
37	**24**·[Ru(η^6-1-Me,3-^{i}Pr-C_6H_4)Cl][PF_6]	1.7°	93.18(2)°	2.5966(7) Å (Sb-Ru)	[2]
38	**24**·[Rh(η^5-C_5Me_5)Cl][PF_6]	2.7°	92.13(1)°	2.5602(5) Å (Sb-Rh)	[2]
39	**24**·$PdCl_2$	1.1°	95.68(1)°	2.5020(5) Å (Sb-Pd)	[2]
40	(**24**)$_2$·(μ-Pd)(SbF_6)	1.8°	91.06(1)°	2.6142(4) Å (Sb-Pd)	[2]
41	**24**·[Pd(η^2-maleicanhydride)]	2.5°	99.64(1)°	2.5542(4) Å (Sb-Pd)	[2]
42	(**24**)$_2$·(μ-Ag)(ClO_4)	5.3°	101.70(2)° and 103.93(2)°	2.7044(5) Å (Sb-Ag)	[2]
43	**24**·$PtCl_2$	1.0°	96.49(1)°	2.5007(5) Å (Sb-Pt)	[2]
44	(**24**)$_2$·(μ-Au)($AuCl_2$)	2.0°	103.83(1)°	2.6495(5) Å (Sb-Au)	[2]
45	(**24**)$_2$·(μ-Au)(SbF_6)	2.4°	104.34(2)°	2.6355(6) Å (Sb-Au)	[2]
46	**33**·[Ru(η^6-1-Me,3-^{i}Pr-C_6H_4)Cl]	1.4(1)°	88.83(4)°	2.3564(5) Å (Pd-P) and 2.158(1) Å (Pd-N)	[4]
47	**33**·$PdCl_2$	3.6°	88.26(4)°	2.2529(6) Å (Pd-P) and 2.051(2) Å (Pd-N)	[4]
48	(**33**)$_2$·(μ-Pd_2Cl_2)(BARF)$_2^{\$}$	1.9°	93.12(5)°	2.2562(5) Å (Pd-P) and 2.048(2) Å (Pd-N)	[4]
49	**34a**·PdBr(p-CN-C_6H_4)	1.3°	95.04(5)°	2.2607(5) Å (Pd-P) and 2.170(2) Å (Pd-N)	[5]
50	[**34a**·Pd(p-CN-C_6H_4)]$_n$(SbF_6)$_n$	3.0°	96.28(9)°	2.247(1) Å (Pd-P) and 2.166(3) Å (Pd-N)	[5]
51	(**34a**)$_2$·(μ-$Pd_2(OH)_2$)(SbF_6)$_2$	2.2°	93.73(5)°	2.2266(6) Å (Pd-P) and 2.038(2) Å (Pd-N)	[5]
52	[**34a**·Pd(acac)](SbF_6)	3.1°	90.87(5)	2.2615(6) Å (Pd-P) and 2.039(2) Å (Pd-N)	[5]
53	**34a**·$PtCl_2$	4.9°	92.4(2)°	2.243(2) Å (Pt-P) and 2.042(5) Å (Pt-N)	[5]
54	(**34a**)$_2$·(μ-Pt_2Cl_2)(BARF)$_2^{\$}$	1.5°	93.73(8)°	2.2370(8) Å (Pt-P) and 2.029(5) Å (Pt-N)	[5]

Double chelated complex (**G**, Figure 2)

55	**17j**·($PdCl_2$)$_2$	9.1°	82.27(7)°	1.447(3) Å (N-Pd)	[11]

Annellated species with transition metal bridges (**H**, Figure 2)

56	(**11d**-2H)Zr(NMe_2)$_2$	6.4(3)°	104.68(15)°	2.081(3) Å (N-Zr)	[14]
57	(**11d**-2H)Zr(CH_2Ph)$_2$	5.9(2)°	112.94(11)°	2.0523(19) Å (N-Zr)	[14]

Multidentate chelated species with main-group elements, transition metals and lanthanide bridges [**I** (Entries 58-78) and **J** (Entries 79-81), Figure 2]

58	(**9d**-2H)[C-RhCl(cod)]$^{¢}$	17.4°	121.49°	1.370 Å (N-C)	[15]
59	(**9d**-2H)[C-Mo(CO)$_4$]	15.5°	122.80°	1.339 Å (N-C)	[15]
60	(**9e**-2H)Mg(THF)$_2$	2.9°	123.18(7)°	1.983(2) Å (N-Mg)	[16]
61	(**9e**-2H)$TiCl_2$	4.3°	134.01(9)°	1.891(2) Å (N-Ti)	[16]
62	(**9e**-2H)$TiMe_2$	4.1°	134.71(7)°	1.921(2) Å (N-Ti)	[16]
63	(**9e**-2H)$ZrBz_2$	10.1°	138.6(1)°	2.082(3) Å (N-Zr)	[16]
64	(**18a**-2H)Mg(THF)$_2$	4.3°	101.3°	1.974(5) Å (O^{Ph}-Mg) and 2.238(6) Å (N-Mg)	[17]
65	(**18c**-2H)$ZrBz_2$	3.9°	103.3(1)°	2.013(3) Å (O^{Ph}-Zr) and 2.356(4) Å (N-Zr)	[17]
66	(**18e**-2H)AlO^{i}Pr	NR	NR	NR	[18]
67	(**18e**-2H)Ti(O^{i}Pr)$_2$	5.1°	87.77°	1.977(3) Å (O^{Ph}-Ti) and 2.214(3) Å (N-Ti)	[19]
68	(**18e**-2H)Co	6.7°	128.96(9)°	1.910(2) Å (O^{Ph}-Co) and 2.006(2) Å (N-Co)	[20]
69	[(**18e**-2H)Co](BARF)$^{\$}$	8.3°	108.63(6)°	1.8845(13) Å (O^{Ph}-Co) and 1.9984(15) Å (N-Co)	[20]
70	(**18e**-2H)Y(O^{t}Bu)THF	4.1°	104.4(1)°	2.170 Å (O^{Ph}-Y) and 2.426 Å (N-Y)	[21]
71	(**18e**-2H)Zr(O^{n}Pr)$_2$	5.8°	96.93°	2.043(3) Å (O^{Ph}-Zr) and 2.373(4) Å (N-Zr)	[22]
72	(**18e**-2H)Zr(O^{i}Pr)$_2$	5.2°	98.26°	2.031 Å (O^{Ph}-Zr) and 2.367 Å (N-Zr)	[22]
73	(**18e**-2H)Zr(O^{t}Bu)$_2$	3.1°	85.90°	2.120(1) Å (O^{Ph}-Zr) and 2.349(2) Å (N-Zr)	[22]
74	(**18e**-2H)In(Cl)THF	4.1°	100.15(6)°	2.102 Å (O^{Ph}-In) and 2.248 Å (N-In)	[23]
75	(**18e**-2H)Ce(O^{t}Bu)$_2$	12.6°	114.6(1)°	2.221 Å (O^{Ph}-Ce) and 2.484 Å (N-Ce)	[21]

76	(**19a**-2H)Ce(O^{t}Bu)$_2$	1.9°	100.15°	2.2670(10) Å (O^{Ph}-Ce) and 2.5183(12) Å (N-Ce)	[24]
77	(**19b**-2H)Y(CH$_2$SiMe$_3$)	3.9°	85.92(8)°	2.128(2) Å (O^{Ph}-Y) and 2.429(2) Å (N-Y)	[25]
78	(**19b**-2H)YBz	1.8°	88.25(11)°	2.139(3) Å (O^{Ph}-Y) and 2.388(3) Å (N-Y)	[25]
79	(**34a**-H)·PdCl	1.6°	98.51(4)° (P-Pd-N^{Cp}) and 163.57(4)° (P-Pd-N^{Gdn})¥	2.2442(5) Å (Pd-P) and 2.061(1) Å (Pd-N^{Cp})	[5]
80	[(**34a**-H)·Pd(dmap)](SbF$_6$)	1.5°	97.43(4)° (P-Pd-N^{Cp}) and 162.49(5)° (P-Pd-N^{Gdn})¥	2.2601(6) Å (Pd-P) and 2.046(1) Å (Pd-N^{Cp})	[5]
81	[(**34a**-H)·Pd(2-PhPy)](SbF$_6$)	3.9°	97.56(6)° (P-Pd-N^{Cp}) and 162.85(7)° (P-Pd-N^{Gdn})¥	2.2529(6) Å (Pd-P) and 2.027(2) Å (Pd-N^{Cp})	[5]
Higher order species with intermolecular N-M-N bridges (M = Co, Zn) (**K**, Figure 2)					
82	[(**18e**-2H)Co]$_4$	NA	NA	1.9005(15) Å (O^{Ph}-Co) and 1.9866(18) Å (N-Co)	[20]
83	[(**18e**-2H)Zn]$_4$	NA	NA	1.906(2) Å (O^{Ph}-Zn) and 2.009(2) Å (N-Zn)	[20]
Carbenes, silylenes, germylenes, and stannylenes of 1,1′-diaminoferrocenes (**L**, Figure 2)					
84	(**9a**-2H)Ge	6.5°	107.44(16) °	1.861(4) Å (N-Ge)	[26]
85	(**9a**-2H)Ge(SePh)$_2$	5.8°	114.9(4)°	1.843(6) Å (N-Ge)	[27]
86	(**9a**-2H)Sn	3.0°	103.2(2)°	2.066(4) Å (N-Sn)	[26]
87	(**9b**-2H)C	16.8°	120.5°	1.361(2) Å (N-C)	[28]
88	[(**9b**-2H)CH][BF$_4$]	16.1°	129.7(2)°	1.331(3) Å (N-C)	[28]
89	(**9b**-2H)[C-RhCl(cod)]¢	17.9°	122.98°	1.374(7) (N-C)	[28]
90	(**9b**-2H)Ge	6.5°	106.7(2)°	1.852(5) Å (N-Ge)	[29]
91	(**9b**-2H)$_2$Sn	1.9°	112.28°	2.045 Å (N-Sn)	[29]
92	[(**9d**-2H)CH][BF$_4$]	15.4°	131.1(6)°	1.330 Å (N-C)	[15]
93	(**9d**-2H)C	16.5°	119.2(7)°	1.36(1) Å (N-C)	[15]
94	(**9d**-2H)Ge	7.1°	105.4(4)°	1.848(2) Å (N-Ge)	[27]
95	[(**9d**-2H)Ge(μ-S)]$_2$	5.5°	111.75(16)°	1.863(3) Å (N-Ge)	[27]
96	[(**9d**-2H)Ge(μ-Se)]$_2$	6.1°	111.1(2)	1.847(5) Å (N-Ge)	[27]
97	(**9d**-2H)(Ge$_3$OCl$_2$)	7.4°	82.40(13)-83.19(13)°	2.073(4) Å and 2.098(4) Å (N-Ge)	[26]
98	(**9e**-2H)Ge	8.3°	106.48(13)°	1.845(2) Å (N-Ge)	[29]
99	(**9e**-2H)Ge(SePh)$_2$	6.4°	115.34(11)°	1.831(2) Å (N-Ge)	[27]
100	(**9e**-2H)Sn	5.1°	103.1(3)°	2.058(6) Å (N-Sn)	[29]
101	(**10**-2H)(CH)(BF$_4$)	15.7°	129.6(3)°	1.327(4) Å (N-C)	[30]
102	(**10**-2H)[C(cod)Cl]¢	18.3°	120.7(3)°	1.359(5) Å (N-C)	[30]
103	(**10**-2H)[C(CO)$_2$Cl]	17.0°	120.4(3)°	1.348(4) Å (N-C)	[30]
104	(**11b**-2H)Si(SePh)$_2$	8.0°	112.46(14)°	1.736(3) Å (N-Si)	[31]
105	(**11b**-2H)Ge	8.1°	103.7(2)°	1.850(6) Å (N-Ge)	[29, 32]
106	(**11b**-2H)Ge(SePh)$_2$	6.5°	106.24(14)°	1.866(5) Å (N-Ge)	[27]
107	[(**11b**-2H)Ge(μ-Se)]$_2$	7.5	108.41(10)°	1.837(2) Å (N-Ge)	[27]
108	[(**11b**-2H)Ge]$_2$·Mo(CO)$_4$	10.2°	104.8(7)°	1.810(11) Å (N-Ge)	[32]
109	(**11b**-2H)Sn	5.6°	100.6(1)°	2.059(3) Å (N-Sn)	[29]
110	(**11c**-2H)Si	8.3°	106.58(6)°	1.733(1) Å (N-Si)	[31]
111	[(**11c**-2H)SiO]$_2$	16.4°	107.24(12)°	1.733 Å (N-Si)	[31]
112	[(**11c**-2H)SiO$_2$]$_2$C	10.3°	112.7(2)°	1.695(5) Å (N-Si)	[31]
113	(**11c**-2H)Si(SePh)$_2$	11.0°	109.49(9)°	1.745(2) Å (N-Si)	[31]

114	(**11c**-2H)Si(H)(OH)	8.4°	111.49(8)°	1.720(2) Å (N-Si)	[31]
115	(**11c**-2H)Si(H)(NH_2)	6.5°	111.7(1)°	1.721(3) Å (N-Si)	[31]
116	(**11c**-2H)Si(H)(PHFc)	7.0°	110.3(3)°	1.735(8) Å (N-Si)	[31]
117	(**11c**-2H)Si(H)(BH_2NH_3)	6.2°	107.84(7)°	1.757(2) Å (N-Si)	[31]
118	(**11c**-2H)Ge	7.6°	103.51(13)°	1.853(3) Å (N-Ge)	[27]
119	[(**11c**-2H)Ge(μ-Se)]$_2$	10.8°	106.21(12)°	1.856(3) Å (N-Ge)	[27]
120	(**11c**-2H)Ge(SePh)$_2$·1/2C_6H_6	8.6° and 9.2°	107.5(3)° and 108.4(3)°	1.847(6) Å and 1.851(6) Å (N-Ge)	[27]
Compounds obtained by oxidation from ***24*** (**M** and **N**, Figure 2)					
121	**24**(F)$_2$	NA	NA	1.959(1) Å (Sb-F)	[2]
122	**24**(F)$_4$	NA	NA	1.977(1) Å (Sb-F)	[2]
123	**24**(Cl)$_4$	NA	NA	2.4428(6) Å (Sb-Cl)	[2]
124	($SbPh_2F$)$_2$O[3]FCP (*i.e.* μ-(Sb-O-Sb)-**24**(F)$_2$O)	2.1°	143.9(7)°	1.982(1) Å (Sb-O) and 2.026(1) Å (Sb-F)	[2]
125	($SbPh_2Cl$)$_2$O[3]FCP (*i.e.* μ-(Sb-O-Sb)-**24**(Cl)$_2$O)	4.0°	144.5(2)°	1.969(3) Å (Sb-O) and 2.563(1) Å (Sb-Cl)	[2]
126	($SbPh_2ONO_2$)$_2$O[3]FCP (*i.e.* μ-(Sb-O-Sb)-**24**(ONO_2)$_2$O)	2.0°	141.81(8)°	1.955(2) Å (Sb-$O^{bridged}$) and 2.304(2) Å (Sb-$O^{terminal}$)	[2]
127	($SbPh_2OClO_3$)$_2$O[3]FCP (*i.e.* μ-(Sb-O-Sb)-**24**($OClO_3$)$_2$O)	1.1°	140.41(9)°	1.942(2) Å (Sb-$O^{bridged}$) and 2.423(2) Å (Sb-$O^{terminal}$)	[2]

* Values for α, reported with no standard deviations, were calculated via Mercury by the authors of this article.[33, 34]
\# NA = Not applicable and NR = Not recorded.
$ BARF = B(3,5-(CF_3)$_2C_6H_3$)$_4$
¢ cod = 1,5-cyclooctadiene
¤ Im = 1,3-di(propan-2-yl)-1,3-dihydro-2H-imidazol-2-ylidene
¥ GDN = guanidine

Table S2. Molecular parameters of cationic Ni(II), Pd(II), Sc(III), Lu(III) complexes of dppf and its 1,1'-diamino-, selected 1,1'-bisphosphanyl- and 1,1'-aminophosphanylferrocenes with Fe→Pd bonding interactions.

	Square-planar Pd(II) complexes	Avg. $C^{ipso,Cp}$-E Bond Length	Ni/Pd-Fe distance	Tilt Angle (α)*	Bite Angle (β_n)	Ref.
Cationic Pd(II) complexes with dppf-analogs for comparison						
1	[dppf·Pd(PPh_3)][BF_4]$_2$	1.787 Å[a] (E = P)	2.8934 Å[a]	19.7°[a]	156.79°[a]	[35, 36]
2	[dppf·PdP(C_5H_4-*p*-F)$_3$][BF_4]$_2$	1.784 Å (E = P)	3.0014(4) Å	22.1°	157.54(3)°	[37]
3	[Fc'($PMes_2$)(P^tBu_2)·PdCl][SbF_6][a]	1.795 (E = P^{tBu})[a] and 1.788 Å (E = P^{Mes})[a]	2.8369(10) and 2.7974(10) Å	21.5°[a]	161.60(5)° and 161.46(5)°	[8]
Cationic Pd(II) complexes for 1,1'-diaza- and 1,1'-azaphosphaferrocenes						
4	[**17c**·PdMe][BPh_4]	1.406(4) Å (E = N)	2.7805(5) Å	14.1°	161.9(1)°	[38]
5	[**17e**·Pd(THF)][BF_4]$_2$	1.393(6) Å (E = N)	2.6493(8) Å	18.6°	162.4(1)°	[38]
6	[**17e**·PdCl]Cl	1.394(6) Å (E = N)	2.67 Å	17.7°	162.07(14)°	[39]
7	[**17e**·PdMe][BPh_4]	1.408(3) Å (E = N)	2.7954(5) Å	13.5°	160.29(6)°	[38]
8	[**17f**·PdCl][$PdCl_4$]	1.346(7) Å (E = N)	2.7136(10) Å	17.1°	161.5(2)°	[40]
9	[**17g**·PdCl][Pd(μ-Cl)Cl_2]$_2$	1.425(13) Å (E = N)	2.6814(16) Å	15.0°	160.4(3)°	[40]
10	[**17h**·PdCl][$PdCl_4$]	1.392(7) Å (E = N)	2.6884(8) Å	15.6°	161.0(2)°	[40]
11	[**17i**·PdCl]Cl	1.380(4) Å (E = N)	2.6509(5) Å	16.5°	164.9(1)°	[12]
12	[**17i**·PdCl][$PdCl_4$]	1.379(4) Å (E = N)	2.6616(6) Å	15.6°	164.7(2)°	[12]
13	[**17i**·PdCl][BF_4]	1.380(2) Å (E = N)	2.6447(3) Å	17.6°	165.42(6)°	[12]

14	[**17i**·PdMe][BF_4]	1.390(3) Å (E = N)	2.7475(4) Å	16.5°	160.56(8)°	[12]
15	[**17i**·Pd(PPh_3)][BF_4]$_2$	1.396(3) Å (E = N)	2.7424(3) Å	16.7°	157.49(7)°	[12]
16	[**17i**·Pd(NCMe)][BF_4]$_2$	1.379(3) Å (E = N)	2.6297(4) Å	18.3°	164.39(9)°	[12]
17	[**28a**·PdCl][SbF_6]$_2$	1.783(11) (E = P) and 1.394(15) Å (E = N)	2.7384(18) Å	23.3°	163.9(2)°	[8]
18	[**28a**·Pd($PPh_2C_5H_5$)][SbF_6]$_2$	1.788(8) (E = P) and 1.420(9) Å (E = N)	2.811(3) Å	21.9°	159.11(19)°	[8]
19	[**28a**·Pd(PPh_3)][BF_4]$_2$	1.758(10) (E = P) and 1.415(12) Å (E = N)	2.8289(19) Å	20.9°	158.9(2)°	[8]
20	[**28a**·Pd(PPh_2)Fc'(NMe_2)][BF_4]$_2$	1.766(6) (E = P) and 1.427(7) Å (E = N)	2.8184(9) Å	19.5°	158.79(13)°	[8]
21	[**28a**·PdP(*p*-OMe-C_6H_4)$_3$][BF_4]$_2$	1.770(7) (E = P) and 1.408(9) Å (E = N)	2.8349(11) Å	22.5°	160.45(16)°	[8]
22	[**31**·Pd][SbF_6]$_2$	1.790(7) (E = P) and 1.419(9) Å (E = N)	2.7889(9) Å	21.3°	164.4(2)°	[41]
23	[**34a**·PdCl][SbF_6]	1.775(2) (E = P) and 1.379(3) Å (E = N)	2.7590(5) Å	24.6°	163.01(5)°	[42]
24	[**34b**·PdCl][SbF_6][c]	1.773(2) (E = P) and 1.388(2) Å (E = N)	2.7956(5) Å	22.8°	162.46(5)°	[42]
25	[**34c**·PdCl][SbF_6][d]	1.770(2) (E = P) and 1.384(2) Å (E = N)	2.7821(5) Å	23.0°	163.15(5)°	[42]
Cationic Ni(II) complexes for comparison dppf-analogs for comparison						
26	($C_5H_4P^iPr_2$)Fe·(NiArCl) (Ar = Napthyl)	1.816(3) Å (E = P)	3.498 Å	13.1°	146.04(4)°	[43]
27	($C_5H_4PCy_2$)Fe·(NiArCl) (Ar = Napthyl)	1.813(7) Å (E = P)	3.498 Å	13.1°	146.08(8)°	[43]
Cationic Ni(II) complexes						
28	[**17e**·NiPh][BPh_4]	1.418(4) Å (E = N)	2.8244(6) Å	8.8°	160.44(9)°	[38]
29	[**17i**·Ni(PPh_3)][BF_4]$_2$	1.402(3) Å (E = N)	2.7376(4) Å	11.9°	163.00(8)°	[12]
30	[**17i**·Ni(NCMe)][BF_4]$_2$	1.385(2) Å (E = N)	2.6268(4) Å	13.0°	166.10(7)°	[12]
Complexes with Lu (III), Sc (III), Y (III), La (III)						
31	[(**9d**-2H)Lu(CH_2Ar)(DME)][e]	1.392(6) Å (E = N)	3.342 Å	6.7°	103.7(1)°	[44]
32	[(**9f**-2H)Sc(CH_2Ar)(THF)][e]	1.366(8) Å (E = N)	3.158(2) Å	11.0°	140.9(2)°	[45]
33	[(**9f**-2H)Y(CH_2Ar)(THF)][e]	1.401(7) Å (E = N)	3.240(1) Å	13.2°	133.2(2)°	[46]
34	[(**9f**-2H)La(CH_2Ar)(THF)][e]	1.393(6) Å (E = N)	3.3857(8) Å	12.9°	125.9(1)°	[47]

* Angles, calculated using Mercury as crystallographic software by the authors of this report[33, 34]

[‡] TFAB = tetrakis(pentafluorophenyl)borate,[48] [$B(C_6F_5)_4$]$^-$

[a] Averages from all crystallographically independent entities in the unit cell (standard deviations are excluded)

[b] Im = 1,3-di(propan-2-yl)-1,3-dihydro-2H-imidazol-2-ylidene

[c] Cy = C_6H_{11}

[d] Xyl = 2,4,6-Me_3-C_6H_2

[e] Ar = 3,5-$Me_2C_6H_3$

Table S3. Survey of oxidation potentials (EO) for 1,1'-bispnictogen substituted dppf analogs and their complexes with potentials converted to $(C_5Me_5)_2Fe/(C_5Me_5)_2Fe^+$ scale.[a]

	Compounds and complexes	Solvents for measurements	E^O (V) (as reported)	Internal references during measurements	E^O (V, converted to $(C_5Me_5)_2Fe/(C_5Me_5)_2Fe^+$)*
Oxidation potentials (E^O) for comparison					
1	Ferrocene	CH_3CN	0.40 [1]	$(C_5Me_5)_2Fe/(C_5Me_5)_2Fe^+$	0.40
2	dppf	DCM	-0.08 [49]	Fc/Fc^+	0.32

3	dppf·$PdCl_2$	DCM	0.58 [50]	$(C_5Me_5)_2Fe/(C_5Me_5)_2Fe^+$	0.58
4	Fc'($PMes_2$)(P^tBu_2) ·$PdCl_2$	DCM	0.15 [50]	$(C_5Me_5)_2Fe/(C_5Me_5)_2Fe^+$	0.15

Oxidation potentials (E^O) for 1,1′-bispnictogen substituted dppf analogs

5	**1**	CH_3CN	-0.23 [1]	$(C_5Me_5)_2Fe/(C_5Me_5)_2Fe^+$	-0.23
6	**2**	CH_3CN	-0.40 [51]	Fc/Fc^+	0
7	**4**	DCM	-0.82 [52]	Fc/Fc^+	-0.42
8	**4**	CH_3CN	-0.86 [52]	Fc/Fc^+	-0.46
9	**5**	CH_3CN	-0.10 [1]	$(C_5Me_5)_2Fe/(C_5Me_5)_2Fe^+$	-0.10
10	**6**	CH_3CN	-0.20 [1]	$(C_5Me_5)_2Fe/(C_5Me_5)_2Fe^+$	-0.20
11	**7**	CH_3CN	-0.05 [1]	$(C_5Me_5)_2Fe/(C_5Me_5)_2Fe^+$	-0.05
12	**9b**	DCM	-0.67 [28]	Fc/Fc^+	-0.24
13	**9c**	THF	-0.73 [53]	Fc/Fc^+	-0.33
14	**9d**	THF	-0.69 [53]	Fc/Fc^+	-0.29
15	**9e**	THF	-0.70 [53]	Fc/Fc^+	-0.30
16	**11b**	THF	-0.72 [53]	Fc/Fc^+	-0.32
17	**17a**	DCM	0.52 [10]	Ag/AgCl	0.66
18	**18e**	THF	0.29 [24]	Fc/Fc^+	0.69
19	**19a**	THF	-0.63 [24]	Fc/Fc^+	-0.23
20	**19b**	$C_6H_4F_2$	-0.74 [25]	Fc/Fc^+	-0.34
21	**24**	DCM	0.10 [2]	Fc/Fc^+	0.50
22	**27**	DCM	-0.24 [3]	Fc/Fc^+	0.16
23	**28a**	DCM	0.34 [8]	$(C_5Me_5)_2Fe/(C_5Me_5)_2Fe^+$	0.34
24	**28b**	DCM	0.41 [8]	$(C_5Me_5)_2Fe/(C_5Me_5)_2Fe^+$	0.41
25	**32a**	DCM	0.38 [3]	Fc/Fc^+	0.78
26	**33**	DCM	-0.19 [4]	Fc/Fc^+	0.21
27	**34a**	DCM	-0.29 [42]	Fc/Fc^+	0.11

Oxidation potentials (E^O) for coordination complexes

28	**5**·$Zn(CF_3SO_3)_2$	CH_3CN	0.28 [54]	$(C_5Me_5)_2Fe/(C_5Me_5)_2Fe^+$	0.28
29	**5**·$[Zn(CF_3SO_3)_2]_2$	CH_3CN	0.66 [54]	$(C_5Me_5)_2Fe/(C_5Me_5)_2Fe^+$	0.66
30	$5^{Chelate}$·$Zn(CF_3SO_3)_2$	CH_3CN	0.72 [54]	$(C_5Me_5)_2Fe/(C_5Me_5)_2Fe^+$	0.72
31	**5**·$Co(CF_3SO_3)_2$	CH_3CN	0.25 [54]	$(C_5Me_5)_2Fe/(C_5Me_5)_2Fe^+$	0.25
32	**5**·$[Co(CF_3SO_3)_2]_2$	CH_3CN	0.72 [54]	$(C_5Me_5)_2Fe/(C_5Me_5)_2Fe^+$	0.72
33	$5^{Chelate}$·$Co(CF_3SO_3)_2$	CH_3CN	0.75 [54]	$(C_5Me_5)_2Fe/(C_5Me_5)_2Fe^+$	0.75
34	**13**·$Me(PF_6)$	CH_3CN	0.62 [55]	Fc/Fc^+	1.02
35	**17a**·PdMeCl	DCM	0.78 [10]	Ag/AgCl	0.92
36	[**17a**·PdMeCl]BAF	DCM	0.86 [10]	Ag/AgCl	1.00
37	**24**·$PdCl_2$	DCM	0.47 [2]	Fc/Fc^+	0.87
38	**24**·[Pd(η^2-maleicanhydride)]	DCM	0.34 [2]	Fc/Fc^+	0.74
39	**27**·BH_3	DCM	-0.13 [3]	Fc/Fc^+	0.27
40	**28a**·$[Pd(Ph_2P)Fc'(NMe_2)](BF_4)_2$	DCM	1.10 [50]	$(C_5Me_5)_2Fe/(C_5Me_5)_2Fe^+$	1.10
41	**32a**·AuCl	DCM	0.78 [3]	Fc/Fc^+	1.18
42	[**32a**·AuCl]$_2$	DCM	0.76 [3]	Fc/Fc^+	1.16
43	[**32a**·μ-Au]$_2X_2$ ($X = SbF_6$, NTf_2)	DCM	0.79 [3]	Fc/Fc^+	1.19
44	**33**·$PdCl_2$	DCM	0.26 [4]	Fc/Fc^+	0.66
45	**34a**·$PdCl_2$	DCM	0.22 [42]	Fc/Fc^+	0.62
46	**34a**·$PdCl(SbF_6)$	DCM	0.86 [42]	Fc/Fc^+	1.26
47	**35c**·$PdCl_2$	DCM	0.28 [50]	$(C_5Me_5)_2Fe/(C_5Me_5)_2Fe^+$	0.28
48	**36**·$PdCl_2$	DCM	0.35 [50]	$(C_5Me_5)_2Fe/(C_5Me_5)_2Fe^+$	0.35
49	**37a**·$PdCl_2$	DCM	0.48 [50]	$(C_5Me_5)_2Fe/(C_5Me_5)_2Fe^+$	0.48

Oxidation potentials (E^O) for compounds with metal bridges, carbenes and germylenes

50	[**9b**-2H]$CH[BF_4]$	DCM	0.63 [28]	Fc/Fc^+	1.03

51	[**9b**-2H]C	THF	-0.42 [28]	Fc/Fc$^+$	0.02
52	[**9b**-2H][C-RhCl(CO)$_2$]	DCM	0.46 [28]	Fc/Fc$^+$	0.86
53	[**9b**-2H]Ge	THF	-0.08 [29]	Fc/Fc$^+$	0.32
54	[**9c**-2H]Ge	THF	-0.13 [32]	Fc/Fc$^+$	0.28
55	[**9d**-2H]C	DCM	-0.42 [28]	Fc/Fc$^+$	0.02
56	[**9e**-2H]Ge	THF	-0.13 [29]	Fc/Fc$^+$	0.27
57	[**11b**-2H]Ge	THF	0.15 [29, 32]	Fc/Fc$^+$	0.55
58	(**14**-2H)(O)$_2$	DCM	0.56 [56]	$(C_5Me_5)_2Fe/(C_5Me_5)_2Fe^+$	0.56
59	(**14**-2H)[IrCl(cod)]$_2$	DCM	0.75 [56]	$(C_5Me_5)_2Fe/(C_5Me_5)_2Fe^+$	0.75
60	(**14**-2H)[IrCl(CO)$_2$]$_2$	DCM	0.89 [56]	$(C_5Me_5)_2Fe/(C_5Me_5)_2Fe^+$	0.89
61	(**18e**-2H)AlO^{i}Pr	*o*-difluorobenzene	0.16 [18]	Fc/Fc$^+$	0.56
62	(**18e**-2H)Zn	DCM	0.19 [20]	Fc/Fc$^+$	0.59
63	(**18e**-2H)Co	DCM	0.22 [20]	Fc/Fc$^+$	0.62
64	(**18e**-2H)Y(O^{t}Bu)THF	THF	0.09 [24]	Fc/Fc$^+$	0.49
65	(**18e**-2H)Ce(O^{t}Bu)$_2$	THF	-0.28 [24]	Fc/Fc$^+$	0.12
66	(**18e**-2H)Ce(O^{t}Bu)THF	THF	-0.21 [24]	Fc/Fc$^+$	0.19
67	(**19a**-2H)Y(O^{t}Bu)	THF	-0.29 [24]	Fc/Fc$^+$	0.11
68	(**19a**-2H)Ce(O^{t}Bu)THF	THF	-0.57 [24]	Fc/Fc$^+$	-0.17
69	(**19a**-2H)Ce(O^{t}Bu)$_2$	THF	-0.38 [24]	Fc/Fc$^+$	0.02
70	(**19b**-2H)YCl	$C_6H_4F_2$	-0.22 [25]	Fc/Fc$^+$	0.18
71	(**19b**-2H)Y(CH$_2$Ph)	$C_6H_4F_2$	-0.39 [25]	Fc/Fc$^+$	0.01
Compounds obtained by oxidation of **24**					
72	**24**·(Cl)$_4$	DCM	0.54 [2]	Fc/Fc$^+$	0.94

* For easy comparison, E^O values of all the complexes were converted into the scale of $(C_5Me_5)_2Fe/(C_5Me_5)_2Fe^+$ internal reference by following E^O (V, ref: $(C_5Me_5)_2Fe/(C_5Me_5)_2Fe^+$) = E^O (V, ref: Fc/Fc$^+$) + 0.40 and E^O (V, ref: $(C_5Me_5)_2Fe/(C_5Me_5)_2Fe^+$) = E^O_{DCM} (V, ref: Ag/Ag$^+$) – 0.26 + 0.40.[57, 58]

$ BARF = B(3,5-$(CF_3)_2C_6H_3)_4$

References

1. Plenio, H.; Burth, D., Aminoferrocenes and Aminocobaltocenes as Redox-Active Chelating Ligands: Syntheses, Structures, and Coordination Chemistry1. *Organometallics* **1996,** 15, (19), 4054-4062.
2. Schulz, J.; Antala, J.; Cisarova, I.; Stepnicka, P., Beyond phosphorus: synthesis, reactivity, coordination behaviour and catalytic properties of 1,1'-bis(diphenylstibino)ferrocene. *Dalton Trans.* **2023,** 52, (5), 1198-1211.
3. Skoch, K.; Cisarova, I.; Schulz, J.; Siemeling, U.; Stepnicka, P., Synthesis and characterization of 1'-(diphenylphosphino)-1-isocyanoferrocene, an organometallic ligand combining two different soft donor moieties, and its Group 11 metal complexes. *Dalton Trans.* **2017,** 46, (31), 10339-10354.
4. Binnani, C.; Leitner, Z.; Císařová, I.; Štěpnička, P., Synthesis and Coordination Behavior of a Hybrid Phosphanylferrocene Amidine Ligand. *Eur. J. Inorg. Chem.* **2024,** 27, (3), e202300644.
5. Bárta, O.; Císařová, I.; Štěpnička, P., The protonation state governs the coordination of phosphinoferrocene guanidines. *Dalton Trans.* **2021,** 50, (41), 14662-14671.
6. Song, L.-C.; Wang, G.-F.; Liu, P.-C.; Hu, Q.-M., Synthetic and Structural Studies on Transition Metal Fullerene Complexes Containing Phosphorus and Arsenic Ligands: Crystal and Molecular Structures of (η^2-C_{60})M(dppf) (dppf = 1,1′-Bis(diphenylphosphino)ferrocene; M = Pt, Pd), (η^2-C_{60})Pt(AsPh$_3$)$_2$, (η^2-C_{60})Pt(dpaf) (dpaf = 1,1′-Bis(diphenylarsino)ferrocene), and (η^2-C_{70})Pt(dpaf). *Organometallics* **2003,** 22, (22), 4593-4598.
7. de Lima, G. M.; Filgueiras, C. A. L.; Giotto, M. T. S.; Mascarenhas, Y. P., Tin, palladium and platinum derivatives of 1,1′-bis(diphenylphosphine)ferrocene. Crystal and molecular structures of 1,1′-bis-(diphenylphosphine)ferrocenedichloropalladium(II) and of 1,1′-bis(diphenylphosphine)ferrocenedichloroplatinum(II). *Transition Met. Chem.* **1995,** 20, (4), 380-383.
8. Dey, S.; Roesler, F.; Bruhn, C.; Kelemen, Z.; Pietschnig, R., Tailoring the Fe → Pd interaction in cationic Pd(ii) complexes via structural variation of the ligand scaffold of sterically demanding dppf-analogs and their P,N-counterparts. *Inorg. Chem. Front.* **2023,** 10, 3828-3843.
9. Weng, Z.; Koh, L. L.; Hor, T. S. A., Suzuki cross-coupling in aqueous media catalyzed by a 1,1′-N-substituted ferrocenediyl Pd(II) complex. *J. Organomet. Chem.* **2004,** 689, (1), 18-24.

10. Gibson, V. C.; Gregson, C. K. A.; Halliwell, C. M.; Long, N. J.; Oxford, P. J.; White, A. J. P.; Williams, D. J., The synthesis, coordination chemistry and ethylene polymerisation activity of ferrocenediyl nitrogen-substituted ligands and their metal complexes. *J. Organomet. Chem.* **2005,** 690, (26), 6271-6283.
11. Jess, K.; Baabe, D.; Freytag, M.; Jones, P. G.; Tamm, M., Transition-Metal Complexes with Ferrocene-Bridged Bis(imidazolin-2-imine) and Bis(diaminocyclopropenimine) Ligands. *Eur. J. Inorg. Chem.* **2017,** 2017, (2), 412-423.
12. Jess, K.; Baabe, D.; Bannenberg, T.; Brandhorst, K.; Freytag, M.; Jones, P. G.; Tamm, M., Ni–Fe and Pd–Fe Interactions in Nickel(II) and Palladium(II) Complexes of a Ferrocene-Bridged Bis(imidazolin-2-imine) Ligand. *Inorg. Chem.* **2015,** 54, (24), 12032-12045.
13. Fitzpatrick, M. G.; Hanton, L. R.; Simpson, J., Conformationally flexible arsine ligands: two forms of bis [1,1′-bis (dimethylarsino) ferrocene] dibromonickel (II). *Inorg. Chim. Acta* **1996,** 244, (1), 131-136.
14. Siemeling, U.; Auch, T.-C.; Tomm, S.; Fink, H.; Bruhn, C.; Neumann, B.; Stammler, H.-G., Zirconium Chelates of a Bulky Ferrocene-Based Diamido Ligand. *Organometallics* **2007,** 26, (4), 1112-1115.
15. Siemeling, U.; Färber, C.; Bruhn, C., A stable crystalline N-heterocyclic carbene with a 1,1′-ferrocenediyl backbone. *Chem. Commun.* **2009**, (1), 98-100.
16. Shafir, A.; Power, M. P.; Whitener, G. D.; Arnold, J., Silylated 1,1′-Diaminoferrocene: Ti and Zr Complexes of a New Chelating Diamide Ligand. *Organometallics* **2001,** 20, (7), 1365-1369.
17. Shafir, A.; Fiedler, D.; Arnold, J., Formation of 1：1 complexes of ferrocene-containing salen ligands with Mg, Ti and Zr. *J. Chem. Soc., Dalton Trans.* **2002**, (4), 555-560.
18. Lai, A.; Hern, Z. C.; Diaconescu, P. L., Switchable Ring-Opening Polymerization by a Ferrocene Supported Aluminum Complex. *ChemCatChem* **2019,** 11, (16), 4210-4218.
19. Brown, L. A.; Rhinehart, J. L.; Long, B. K., Effects of Ferrocenyl Proximity and Monomer Presence during Oxidation for the Redox-Switchable Polymerization of l-Lactide. *ACS Catal.* **2015,** 5, (10), 6057-6060.
20. Shepard, S. M.; Diaconescu, P. L., Redox-Switchable Hydroelementation of a Cobalt Complex Supported by a Ferrocene-Based Ligand. *Organometallics* **2016,** 35, (15), 2446-2453.
21. Broderick, E. M.; Diaconescu, P. L., Cerium(IV) Catalysts for the Ring-Opening Polymerization of Lactide. *Inorg. Chem.* **2009,** 48, (11), 4701-4706.
22. Dai, R.; Lai, A.; Alexandrova, A. N.; Diaconescu, P. L., Geometry Change in a Series of Zirconium Compounds during Lactide Ring-Opening Polymerization. *Organometallics* **2018,** 37, (21), 4040-4047.
23. Quan, S. M.; Diaconescu, P. L., High activity of an indium alkoxide complex toward ring opening polymerization of cyclic esters. *Chem. Commun.* **2015,** 51, (47), 9643-9646.
24. Broderick, E. M.; Thuy-Boun, P. S.; Guo, N.; Vogel, C. S.; Sutter, J.; Miller, J. T.; Meyer, K.; Diaconescu, P. L., Synthesis and Characterization of Cerium and Yttrium Alkoxide Complexes Supported by Ferrocene-Based Chelating Ligands. *Inorg. Chem.* **2011,** 50, (7), 2870-2877.
25. Brosmer, J. L.; Diaconescu, P. L., Yttrium-Alkyl Complexes Supported by a Ferrocene-Based Phosphinimine Ligand. *Organometallics* **2015,** 34, (11), 2567-2572.
26. Volk, J.; Bicho, B. A. C.; Bruhn, C.; Siemeling, U., N-Heterocyclic germylenes and stannylenes of the type [Fe{(η5-C5H4)NR}2E] with bulky alkyl substituents. *Z. fur Naturforsch. - B J. Chem. Sci.* **2017,** 72, (11), 785-794.
27. Weyer, N.; Guthardt, R.; Correia Bicho, B. A.; Oetzel, J.; Bruhn, C.; Siemeling, U., Stable N-Heterocyclic Germylenes of the Type [Fe{(η5-C5H4)NR}2Ge] and Their Oxidation Reactions with Sulfur, Selenium, and Diphenyl Diselenide. *Z. Anorg. Allg. Chem.* **2019,** 645, (3), 188-197.
28. Siemeling, U.; Färber, C.; Leibold, M.; Bruhn, C.; Mücke, P.; Winter, R. F.; Sarkar, B.; von Hopffgarten, M.; Frenking, G., Six-Membered N-Heterocyclic Carbenes with a 1,1′-Ferrocenediyl Backbone: Bulky Ligands with Strong Electron-Donor Capacity and Unusual Non-Innocent Character. *Eur. J. Inorg. Chem.* **2009,** 2009, (31), 4607-4612.
29. Oetzel, J.; Weyer, N.; Bruhn, C.; Leibold, M.; Gerke, B.; Pöttgen, R.; Maier, M.; Winter, R. F.; Holthausen, M. C.; Siemeling, U., Redox-Active N-Heterocyclic Germylenes and Stannylenes with a Ferrocene-1,1′-diyl Backbone. *Chem. Eur. J.* **2017,** 23, (5), 1187-1199.
30. Khramov, D. M.; Rosen, E. L.; Lynch, V. M.; Bielawski, C. W., Diaminocarbene[3]ferrocenophanes and Their Transition-Metal Complexes. *Angew. Chem., Int. Ed.* **2008,** 47, (12), 2267-2270.
31. Weyer, N.; Heinz, M.; Schweizer, J. I.; Bruhn, C.; Holthausen, M. C.; Siemeling, U., A Stable N-Heterocyclic Silylene with a 1,1′-Ferrocenediyl Backbone. *Angew. Chem., Int. Ed.* **2021,** 60, (5), 2624-2628.
32. Walz, F.; Moos, E.; Garnier, D.; Köppe, R.; Anson, C. E.; Breher, F., A Redox-Switchable Germylene and its Ligating Properties in Selected Transition Metal Complexes. *Chem. Eur. J.* **2017,** 23, (5), 1173-1186.

33. Macrae, C. F.; Edgington, P. R.; McCabe, P.; Pidcock, E.; Shields, G. P.; Taylor, R.; Towler, M.; van de Streek, J., Mercury: visualization and analysis of crystal structures. *J. Appl. Crystallogr.* **2006,** 39, (3), 453-457.
34. Macrae, C. F.; Bruno, I. J.; Chisholm, J. A.; Edgington, P. R.; McCabe, P.; Pidcock, E.; Rodriguez-Monge, L.; Taylor, R.; van de Streek, J.; Wood, P. A., Mercury CSD 2.0– new features for the visualization and investigation of crystal structures. *J. Appl. Crystallogr.* **2008,** 41, (2), 466-470.
35. Sato, M.; Shigeta, H.; Sekino, M.; Akabori, S., Synthesis, some reactions, and molecular structure of the Pd(BF_4)$_2$ complex of 1,1′-bis(diphenylphosphino)ferrocene. *J. Organomet. Chem.* **1993,** 458, (1), 199-204.
36. Gramigna, K. M.; Oria, J. V.; Mandell, C. L.; Tiedemann, M. A.; Dougherty, W. G.; Piro, N. A.; Kassel, W. S.; Chan, B. C.; Diaconescu, P. L.; Nataro, C., Palladium(II) and Platinum(II) Compounds of 1,1′-Bis(phosphino)metallocene (M = Fe, Ru) Ligands with Metal–Metal Interactions. *Organometallics* **2013,** 32, (20), 5966-5979.
37. Cabrera, K. D.; Rowland, A. T.; Szarko, J. M.; Diaconescu, P. L.; Bezpalko, M. W.; Kassel, W. S.; Nataro, C., Monodentate phosphine substitution in [Pd(κ^3-dppf)(PR_3)][BF_4]$_2$ (dppf = 1,1′-bis(diphenylphosphino)ferrocene) compounds. *Dalton Trans.* **2017,** 46, (17), 5702-5710.
38. Abubekerov, M.; Khan, S. I.; Diaconescu, P. L., Ferrocene-bis(phosphinimine) Nickel(II) and Palladium(II) Alkyl Complexes: Influence of the Fe–M (M = Ni and Pd) Interaction on Redox Activity and Olefin Coordination. *Organometallics* **2017,** 36, (22), 4394-4402.
39. Metallinos, C.; Tremblay, D.; Barrett, F. B.; Taylor, N. J., 1,1′-Bis(phosphoranylidenamino)ferrocene palladium(II) complexes: An unusual case of dative Fe→Pd bonding. *J. Organomet. Chem.* **2006,** 691, (9), 2044-2047.
40. Klapp, L. R. R.; Bruhn, C.; Leibold, M.; Siemeling, U., Ferrocene-Based Bis(guanidines): Superbases for Tridentate N,Fe,N-Coordination. *Organometallics* **2013,** 32, (20), 5862-5872.
41. Navrátil, M.; Císařová, I.; Štěpnička, P., Synthesis and coordination of hybrid phosphinoferrocenes with extended donor pendants. *Dalton Trans.* **2022,** 51, 14618-14629.
42. Bárta, O.; Gyepes, R.; Císařová, I.; Alemayehu, A.; Štěpnička, P., Synthesis and study of Fe → Pd interactions in unsymmetric Pd(ii) complexes with phosphinoferrocene guanidine ligands. *Dalton Trans.* **2020,** 49, (14), 4225-4229.
43. Jover, J.; Miloserdov, F. M.; Benet-Buchholz, J.; Grushin, V. V.; Maseras, F., On the Feasibility of Nickel-Catalyzed Trifluoromethylation of Aryl Halides. *Organometallics* **2014,** 33, (22), 6531-6543.
44. Wong, A. W.; Miller, K. L.; Diaconescu, P. L., Reactions of aromatic N-heterocycles with a lutetium benzyl complex supported by a ferrocene-diamide ligand. *Dalton Trans.* **2010,** 39, (29), 6726-6731.
45. Carver, C. T.; Monreal, M. J.; Diaconescu, P. L., Scandium Alkyl Complexes Supported by a Ferrocene Diamide Ligand. *Organometallics* **2008,** 27, (3), 363-370.
46. Carver, C. T.; Diaconescu, P. L., Ring-Opening Reactions of Aromatic N-Heterocycles by Scandium and Yttrium Alkyl Complexes. *J. Am. Chem. Soc.* **2008,** 130, (24), 7558-7559.
47. Miller, K. L.; Williams, B. N.; Benitez, D.; Carver, C. T.; Ogilby, K. R.; Tkatchouk, E.; Goddard, W. A., III; Diaconescu, P. L., Dearomatization Reactions of N-Heterocycles Mediated by Group 3 Complexes. *J. Am. Chem. Soc.* **2010,** 132, (1), 342-355.
48. Blass, B. L.; Sánchez, R. H.; Decker, V. A.; Robinson, M. J.; Piro, N. A.; Kassel, W. S.; Diaconescu, P. L.; Nataro, C., Structural, Computational, and Spectroscopic Investigation of [Pd(κ^3-1,1′-bis(di-tert-butylphosphino)ferrocenediyl)X]$^+$ (X = Cl, Br, I) Compounds. *Organometallics* **2016,** 35, (4), 462-470.
49. Dey, S.; Pietschnig, R., Chemistry of sterically demanding dppf-analogs. *Coord. Chem. Rev.* **2021,** 437, 213850.
50. Škoch, K.; Schulz, J.; Císařová, I.; Štěpnička, P., Pd(II) Complexes with Chelating Phosphinoferrocene Diaminocarbene Ligands: Synthesis, Characterization, and Catalytic Use in Pd-Catalyzed Borylation of Aryl Bromides. *Organometallics* **2019,** 38, (15), 3060-3073.
51. Britton, W. E.; Kashyap, R.; El-Hashash, M.; El-Kady, M.; Herberhold, M., The anomalous electrochemistry of the ferrocenylamines. *Organometallics* **1986,** 5, (5), 1029-1031.
52. Blank, N. F.; Glueck, D. S.; Zakharov, L. N.; Rheingold, A. L.; Saybolt, M. D.; Ghent, B. L.; Nataro, C., Synthesis, Structure, and Electrochemistry of an Electron-Rich Chiral Diaminoferrocene, (S,S)-Bis(2,5-dimethylpyrrolidinyl)ferrocene. *Organometallics* **2005,** 24, (21), 5184-5187.
53. Duhović, S.; Diaconescu, P. L., An experimental and computational study of 1,1′-ferrocene diamines. *Polyhedron* **2013,** 52, 377-388.
54. Plenio, H.; Aberle, C., Coupled Molecular Switches: A Redox-Responsive Ligand and the Redox-Switched Complexation of Metal Ions. *Chem. Eur. J.* **2001,** 7, (20), 4438-4446.

55. Kong, D.; Weng, T.; He, W.; Liu, B.; Jin, S.; Hao, X.; Liu, S., Synthesis, characterization, and electrochemical properties of ferrocenylimidazolium. *J. Organomet. Chem.* **2013,** 727, 19-27.
56. Varnado Jr, C. D.; Lynch, V. M.; Bielawski, C. W., 1,1′-Bis(N-benzimidazolylidene)ferrocene: synthesis and study of a novel ditopic ligand and its transition metal complexes. *Dalton Trans.* **2009**, (35), 7253-7261.
57. Elschenbroich, C., *Organometallics*. Wiley-VCH Verlag GmbH & Co. KGaA, Weinheim: 2006.
58. Noviandri, I.; Brown, K. N.; Fleming, D. S.; Gulyas, P. T.; Lay, P. A.; Masters, A. F.; Phillips, L., The decamethylferrocenium/decamethylferrocene redox couple: A superior redox standard to the ferrocenium/ferrocene redox couple for studying solvent effects on the thermodynamics of electron transfer. *J. Phys. Chem. B* **1999,** 103, (32), 6713-6722.
